# AIDS patients suffer higher risk of advanced knee osteoarthritis progression due to lopinavir-induced Zmpste24 inhibition

**DOI:** 10.1038/s41413-025-00431-2

**Published:** 2025-06-03

**Authors:** Keyu Kong, Li Liu, Renfang Zhang, Yongyun Chang, Yueming Shao, Chen Zhao, Hua Qiao, Minghao Jin, Xuzhuo Chen, Wentao Shi, Xinru Wu, Wenxuan Fan, Yuehao Hu, Kewei Rong, Pu Zhang, Baixing Li, Jingwei Zhang, Peixiang Ma, Xiaoling Zhang, Huiwu Li, Zanjing Zhai

**Affiliations:** 1 Shanghai Key Laboratory of Orthopaedic Implants, Department of Orthopaedic Surgery, Shanghai Ninth People’s Hospital, Shanghai Jiaotong University School of Medicine, Shanghai, China; 2 Department of Infection and Immunity, Shanghai Public Health Clinical Center (SPHCC), Fudan University, Shanghai, China; 3 Shanghai Key Laboratory of Stomatology, Department of Oral Surgery, College of Stomatology, Ninth People’s Hospital, Shanghai Research Institute of Stomatology, National Clinical Research Center of Stomatology, Shanghai Jiao Tong University School of Medicine, Shanghai, China; 4 Department of Biostatistics in Clinical Research Unit, Shanghai Ninth People’s Hospital, Shanghai Jiaotong University School of Medicine, Shanghai, China; 5 Department of Orthopedic Surgery, Xinhua Hospital, School of Medicine, Shanghai Jiao Tong University, Shanghai, China

**Keywords:** Diseases, Pathogenesis

## Abstract

Debate regarding the premature aging of knee joints in acquired immune deficiency syndrome (AIDS) patients has remained contentious, with conjectures pointing towards its correlation with distinct antiviral regimes. Protease inhibitors (PIs) stand as a prominent class of antiviral agents frequently utilized in AIDS management and have been significantly linked to premature senescence. This study aimed to investigate whether PI-containing regimens would accelerate osteoarthritis (OA) development and explore the molecular mechanisms underlying this association. A retrospective cohort of 151 HIV-infected individuals, categorized into PI and non-PI groups, was established. Patients in PI group exhibited lower KOOS and a higher prevalence of radiological knee OA than those in non-PI group. Additionally, 25 anti-HIV drugs were screened and among all antiviral drugs, lopinavir had the most detrimental impact on cartilage anabolism, accelerating cartilage senescence and promoting mouse OA development. Mechanistically, lopinavir accelerated cellular senescence by inhibiting Zmpste24 and interfering nuclear membrane stability, which leads to decreased binding between nuclear membrane-binding protein Usp7 and Mdm2 and activates Usp7/Mdm2/p53 pathway. Zmpste24 overexpression reduces OA severity in mice. These findings suggest that PI-containing regimens accelerate cartilage senescence and OA development through Zmpste24 inhibition, which provides new insights into the selection of HIV regimens.

## Introduction

Osteoarthritis (OA) is one of the most significant causes of disability and functional limitation, accounting for 2% of all years of living with disability worldwide.^1^ Risk factors for OA include increasing age, joint biomechanics, genetic factors, and obesity. Chondrocyte senescence was first found in the cartilage of patients with OA in 2002.^2^ It is associated with an increased secretion of pro-inflammatory proteins such as interleukin-6 (IL-6) and IL-8, called senescence-associated secretory phenotype, and contributes significantly to chronic inflammation.^3–5^ Senescence induces cell cycle arrest, and the clearance of senescent chondrocytes could attenuate the progression of OA in mice.^6,7^

The pathological progression of OA in people living with human immunodeficiency virus (HIV) (PLWH) is intricate and remains poorly understood. With the widespread use of highly active antiretroviral therapy (HAART), HIV has transformed from a rapidly fatal infection into a manageable disease.^8^ As PLWH expect a longer life expectancy, significant concerns have emerged regarding the morbidity of non-AIDS comorbidities such as cancer, diabetes, OA, and hypertension.^9^ PLWH are at a higher risk of developing comorbidities than others.^10^ However, there remains controversy regarding the prevalence of OA among PLWH; Ni et al.’s study indicates that the incidence of OA in PLWH does not differ significantly from that in the general population.^9^ In controversy, Liu et al. found that the knees of PLWH have a more heterogeneous cartilage matrix and severe synovitis in the infrapatellar and suprapatellar fat pads.^11^ Additionally, the incidence of hand OA is higher in patients with HIV infection and metabolic syndrome than in the general population.^12^ We speculate that the discrepancies observed across various studies may be attributed to the intricate medication regimens employed among PLWH, as certain regimens may potentially accelerate the progression of OA within this population. Recent research has focused on the effects of long-term medication on cartilage health.^13,14^

There are multiple antiretroviral therapy schemes, and many studies have focused on their roles in the occurrence of comorbidities. Protease inhibitors (PIs) are a prominent class of antiretrovirals, including lopinavir, ritonavir, atazanavir, and darunavir, which are commonly used in addition to nucleoside analog reverse transcriptase inhibitors.^15^ PIs were reported to induce senescence in human bone marrow mesenchymal stem cells,^16^ impair osteoblast differentiation, and promote the proliferation and activation of osteoclasts.^17–19^ Clinical trials have found that an antiretroviral regimen containing PIs is associated with a steeper decline in bone mineral density (BMD) in the spine and a higher risk of osteoporosis.^20,21^ Furthermore, PIs trigger vascular smooth muscle cell senescence and calcification through the downregulation of Zmpste24,^22,23^ an inner nuclear membrane zinc metalloprotease that plays a vital role in the maturation of Lamin A and is closely related to senescence.^24^ However, the role of PIs in OA and molecular mechanisms underlying the association between PIs use and cell senescence remain unclear.

Most PIs inhibit Zmpste24, leading to the cellular accumulation of prelamin A.^25–27^ Impaired biosynthesis of mature Lamin A is associated with several genetic diseases, such as Hutchinson-Gilford progeria syndrome and mandibuloacral dysplasia.^28^ Zmpste24 mutation causes restrictive dermopathy, a neonatal lethal progeroid disorder.^29^ Zmpste24 knockout mice exhibit spontaneous bone fractures, reduced BMD, and progressive hair loss.^30^ Whether the side effects of PIs on senescence are related to the downregulation of Zmpste24 remains uncertain.^16^

This study aimed to investigate whether PI-containing regimens would accelerate osteoarthritis (OA) development and explore the molecular mechanisms underlying this association.

## Results

### Lopinavir/ritonavir use is associated with a high incidence of early OA syndromes in patients with HIV

To explore the potential association between the use of PIs, particularly lopinavir/ritonavir, and the early development of OA, a retrospective cohort of 151 HIV-infected individuals was established, and the specific screening process is detailed in Fig. S1a. Detailed basic characteristics of both groups are presented in Table S1. As lopinavir/ritonavir is the only PI regimen available in Shanghai, we categorized the patients into PI and non-PI groups based on the use of lopinavir/ritonavir.

Patients in the PI group experienced more severe pain and symptoms and acquired lower scores on sports/recreation function (Sport/Rec) scales than the non-PI group (PI vs non-PI: pain score, 90.6 vs 95.1; symptom score, 88.8 vs 93.0; Activities of Daily Living (ADL) score, 95.4 vs 97.2; Sport/Rec score, 90.5 vs 95.4; Quality of Life (QOL) score, 89.4 vs 92.4). Additionally, the PI group showed a higher prevalence of radiological knee OA than the non-PI group (PI vs non-PI: KL ≥ 1, 25.6% vs 8.2%; KL ≥ 2, 10.3% vs 2.7%) (Table 1 and Fig. S1b–g). Notably, in all five subscores of the KOOS, the difference in scores was greater in PLWH aged > 45 years, whereas it was relatively smaller in PLWH aged < 45 years. Specifically, within the 30–45 age bracket, the KOOS pain score average was 1.9 points lower in the PI group than in the non-PI group. Notably, in the 45–80-year-old group, the PI group had an average score of 8.9 points lower than the non-PI group. After adjusting for confounding factors such as metabolic indicators, age, and duration of HIV infection through multivariable logistic regression and multivariable linear regression, we found a negative association between PI drugs and KOOS and a positive relation between PI drugs and radiographic OA with a KL grade ≥ 1 (Table 2).

**Table 1:** KOOS scores and radiographic scores in the whole cohort and in PI and non-PI groups

Group	Protease Inhibitor (*n* = 78)	non-Protease Inhibitor (*n* = 73)	*P* value
*KOOS Pain, mean (SD)*	90.6 (16.0)	95.1 (9.2)	0.038
*30–45 years (n* = *94)*	94.6 (13.1)	96.5 (9.1)	0.425
*46–80 years (n* = *57)*	83.8 (18.2)	92.7 (8.9)	0.026
*KOOS Symptom, mean (SD)*	88.8 (15.4)	93.0 (9.5)	0.048
*30–45 years*	91.9 (14.5)	94.5 (8.6)	0.308
*46–80 years*	83.6 (15.1)	90.4 (10.5)	0.055
*KOOS ADL, mean (SD)*	95.4 (11.4)	97.2 (5.5)	0.222
*30–45 years*	97.8 (7.3)	98.3 (4.9)	0.706
*46–80 years*	91.3 (15.4)	95.3 (6.1)	0.211
*KOOS Sport/Rec, mean (SD)*	90.5 (17.7)	95.4 (10.4)	0.041
*30–45 years*	93.4 (15.7)	97.7 (8.5)	0.135
*46–80 years*	85.0 (19.6)	91.5 (12.2)	0.148
*KOOS QOL, mean (SD)*	89.4 (20.0)	92.4 (13.2)	0.288
*30–45 years*	92.8 (19.4)	94.5 (12.9)	0.617
*46–80 years*	83.8 (20.1)	88.8 (13.2)	0.274
*Radiological OA*			
*KL* ≥ *1, n (%)*	20 (25.6)	6 (8.2)	0.005
*KL* = *1, n (%)*	12 (15.4)	4 (5.5)	0.064
*KL* ≥ *2, n (%)*	8 (10.3)	2 (2.7)	0.100

*PI* protease inhibitors, *KOOS* knee Injury and Osteoarthritis Outcome Score, *ADL* function in daily activities, *Sport/Rec* function in sports and recreation, *QOL* quality of life

**Table 2:** Multivariate analysis of associations between demographic features, metabolic features, HIV features and KOOS scores, radiological OA in our cohort

Multivariate regression analysis	Dependent variable	Model 1		Model 2		Model 3		Model 4	
		β or OR (95% CI)	*P* value	β or OR (95% CI)	*P* value	β or OR (95% CI)	*P* value	β or OR (95% CI)	*P* value
Linear	*KOOS Pain*	−4.48 (−8.70 to −0.26)	0.038	−4.72 (−8.89 to −0.54)	0.027	−5.06 (−9.49 to −0.63)	0.025	−5.33 (−9.08 to −0.86)	0.020
	*KOOS Symptom*	−4.18 (−8.32 to −0.03)	0.048	−4.30 (−8.51 to −0.08)	0.046	−4.51 (−8.93 to −0.10)	0.045	−4.58 (−9.04 to −0.11)	0.045
	*KOOS ADL*	−1.81 (−4.72 to 1.10)	0.222	−2.13 (−4.96 to 0.69)	0.138	−2.34 (−5.35 to 0.67)	0.127	−2.38 (−5.44 to 0.68)	0.126
	*KOOS Sport/Rec*	−4.90 (−9.60 to −0.20)	0.041	−5.61 (−10.25 to −0.98)	0.018	−6.16 (−11.08 to −1.25)	0.014	−6.22 (−11.20 to −1.24)	0.015
	*KOOS QOL*	−2.96 (−8.45 to 2.53)	0.288	−3.63 (−9.17 to 1.92)	0.198	−3.83 (−9.71 to 2.04)	0.199	−4.05 (−9.97 to 1.87)	0.178
Logistic	*Radiographic OA (KL* ≥ *1)*	3.85 (1.53 to 11.12)	0.007	4.72 (1.63 to 15.86)	0.007	6.38 (1.95 to 25.03)	0.004	6.89 (2.06 to 28.01)	0.003

Model 1 was not adjusted for any covariate. Model 2 was adjusted for age and sex. Model 3 was further adjusted for metabolic features including BMI, triglycerides, total cholesterol, HDL-cho, LDL-cho and glycaemia. Model 4 was adjusted for terms in model 3 and HIV features, including durations of HIV infection, CD4 level and viral load (undetectable or not)

*PI* protease inhibitors, *KOOS* knee Injury and Osteoarthritis Outcome Score, *ADL* function in daily activities, *Sport/Rec* function in sports and recreation, *QOL* quality of life

### Lopinavir accelerates chondrocyte degeneration, senescence and OA progression both in vitro and in vivo

**Fig. 1:**
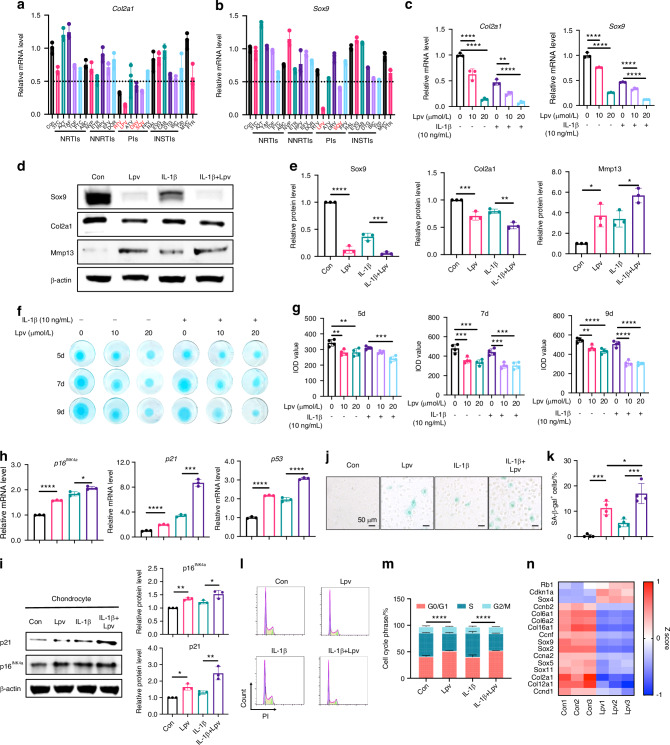
Lopinavir strongly inhibits Col2a1 and Sox9 expression and accelerates chondrocyte senescence and degeneration in vitro. **a**, **b** RT-qPCR of Sox9 and Col2a1 after treatment with anti-HIV drugs; drugs in red boxes indicate >50% reduction. **c** RT-qPCR of *Sox9* and *Col2a1* under IL-1β and varying lopinavir concentrations. **d**, **e** Western blot and quantification of Sox9, Col2a1, and Mmp13 after IL-1β and lopinavir treatment. **f**, **g** Alcian Blue staining and quantification of micromass after IL-1β and lopinavir at different time points. **h** RT-qPCR of senescence-related genes (*p16*^*INK4a*^, *p21*, *p53*) after IL-1β and lopinavir treatment. **i** Western blot and quantification of p16^INK4a^ and p21 in primary chondrocytes. **j**, **k** SA-β-gal staining and quantification in primary chondrocytes. Scale bar: 50 μm. **l**, **m** Cell cycle analysis after IL-1β and lopinavir treatment. **n** Heatmap of cell cycle and chondrogenesis-related gene expression after lopinavir treatment. Data are shown as mean ± SD. *P < 0.05, **P < 0.01, ***P < 0.001, ****P < 0.000 1

The positive association between PI use and the development of OA in PLWH leads to the hypothesis that PIs affect chondrocyte metabolism. To assess the effect of various anti-HIV drugs on chondrocytes, we screened a small-molecule library containing 25 FDA-approved anti-HIV drugs from seven categories (Table S2). After treatment with 20 μmol/L of different drugs, mRNA expression levels of *Col2a1* and *Sox9* in ATDC5 chondrocytes were measured, and we identified four PI-class drugs that reduced the expression of Col2a1 and two PI-class drugs that reduced the expression of Sox9 with 50% reduction threshold (Fig. 1a, b). Among them, lopinavir exhibited the most potent inhibition of both genes (85% for *Col2a1* and 90% for *Sox9*), consistent with our clinical observations.

The administration of 40 μmol/L lopinavir exhibited significant toxicity, while that of 20 μmol/L inhibited cell proliferation, consistent with the results of previous studies.^22^ Therefore, 20 μmol/L concentration was used for subsequent cell treatments (Fig. S2a). We also tested the effects of ritonavir and lopinavir/ritonavir combination on cell proliferation since lopinavir is typically used with ritonavir (Fig. S2b, c). The results showed that 5 μmol/L ritonavir had minimal effect on cell proliferation, indicating that the inhibitory effect of lopinavir/ritonavir on chondrogenesis mainly depends on lopinavir.

In addition, lopinavir reduced mRNA expression of cartilage anabolism-related genes (Col2a1, Sox9) by ~60% and increased the mRNA expression of cartilage catabolism-related genes (*Mmp13*, *Mmp3*, *Mmp9*, *Adamts4*). This inhibitor effect is more evident in the presence of IL-1β (Fig. 1c and Fig. S3a). Meanwhile, protein expression of Col2a1 and Sox9 decreased by 40% and 90% after lopinavir treatment and Mmp13 protein increased by three times (Fig. 1d, e). Similar results were observed when lopinavir was combined with ritonavir (Fig. S3b).

Micromass culture results showed a decrease of IOD value varying from 10% to 40% depending on different culture times, which demonstrated that lopinavir inhibited chondrogenesis, extracellular matrix synthesis, and secretion (Fig. 1f, g, Fig. S3c, d). Additionally, 3D pellet culture showed that lopinavir caused loosening and degeneration of the collagen matrix within the primary chondrocyte pellet by Safranine O, Alcian Blue and Toluidine Blue staining (Fig. S3e). Overexpression of Sox9 significantly rescued lopinavir-induced cartilage degeneration (Fig. S4), indicating that the acceleration of cartilage degeneration by lopinavir depends on the downregulation of Sox9.

Furthermore, the expression of senescence and cell cycle-related markers (p16^INK4a^, p21, and p53) was examined in the lopinavir-treated ATDC5 chondrocytes and primary chondrocytes. The results showed a significant upregulation, from 50% to 100%, of these genes, particularly under inflammatory conditions (Fig. 1h, i, Fig. S3f). Beta-galactosidase staining and cell cycle experiments confirmed that lopinavir induced cell cycle arrest and accelerated chondrocyte senescence (Fig. 1j–m). Transcriptome sequencing was performed on lopinavir-stimulated cells, and gene expression changes related to the cell cycle, chondrogenesis, and matrix synthesis were consistent (Fig. 1n).

**Fig. 2:**
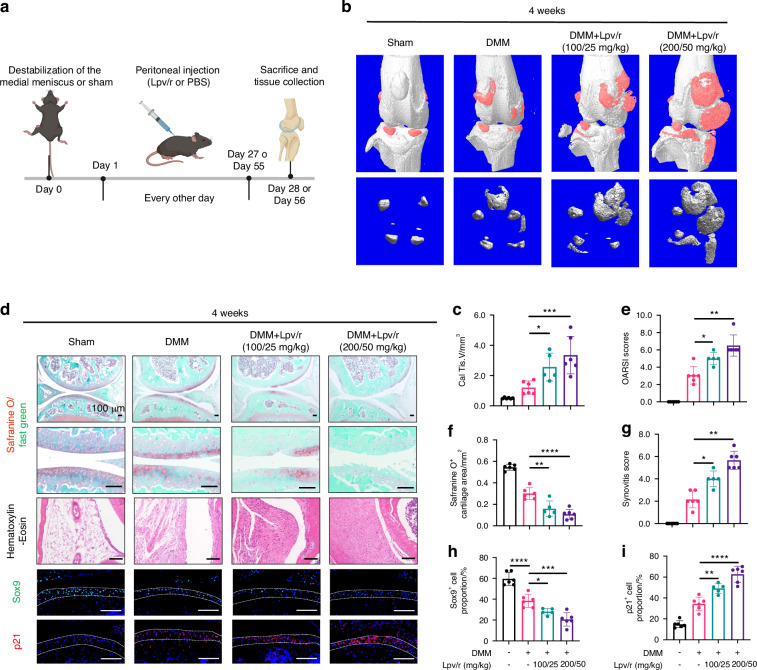
Acceleration of osteoarthritis progression in mice by intraperitoneal injection of lopinavir. **a** Flowchart of animal experiment. **b**, **c** Representative images and quantitative analysis of 3D-reconstructed calcified tissues in different groups of mice at 4 weeks after DMM surgery, *n* = 5 or 6 per group. **d** Representative images of Safranine O staining, HE staining, and immunofluorescence staining for Sox9, Zmpste24, and p21 in different groups of mice 4 weeks after DMM surgery. Scale bar: 100 μm; **e**–**i** Quantitative analysis of OARSI score (**e**), synovial inflammation score (**g**), Safranine O-positive cartilage area (**f**), Sox9-positive cell ratio (**h**) and p21-positive cell ratio (**i**). Data are shown as mean ± SD. *P < 0.05, **P < 0.01, ***P < 0.001, and ****P < 0.000 1

In vivo experiments were conducted in mice receiving destablization of the medial meniscus (DMM) surgery. Lopinavir/ritonavir was administered intraperitoneally every 2 days (Fig. 2a). Four weeks postoperative, DMM caused a decrease in subchondral bone BV/TV, Tb.Th, Tb.N, and lopinavir/ritonavir injection, further exacerbating this decrease in BV/TV and Tb.N by 20% (Fig. S5a, b). At 8 weeks, there was no significant difference in subchondral bone density (Fig. S5c). Additionally, we observed osteophyte formation, cartilage erosion, and synovitis in mice at 4 and 8 weeks after surgery, which were further aggravated by lopinavir/ritonavir injections (Fig. 2b–g and Fig. S6). Immunofluorescence staining of the knee joint cartilage of mice 4 weeks after surgery revealed a significant decrease in the Sox9 expression level. In contrast, the expression level of the senescence marker p21 increased (Fig. 2d, h, i). The intraperitoneal injection of the drug further amplified these trends, decreasing Sox9 positive cell proportion from 36% to 18% and p21 positive cell proportion from 32% to 60%.

Therefore, lopinavir accelerated the degeneration and senescence of chondrocytes and contributed to the occurrence and development of OA both in vitro and in vivo.

### Lopinavir inhibits Zmpste24 activity, and Zmpste24 was downregulated in OA

Previous studies reported that lopinavir inhibits the function of Zmpste24,^26^ a well-known gene associated with senescence. Lopinavir stimulation induced nuclear membrane blebbing through immunofluorescence staining of Zmpste24-processed Lamin A (Fig. S7a, b). This indicates the inhibition of Zmpste24 and decreased stability of the nuclear membrane. A decrease in the expression of nuclear membrane-related genes was observed following lopinavir treatment (Fig. S7c). Decreased Lamin A expression and a prelamin A band provided direct evidence of Zmpste24 inhibition and impaired Lamin A maturation (Fig. S7d, e). The elevation of DNA damage and the presence of heterochromatin markers, such as H3K9ME3 and γH2AX, also suggested compromised nuclear membrane and chromatin stability (Fig. S7f).

**Fig. 3:**
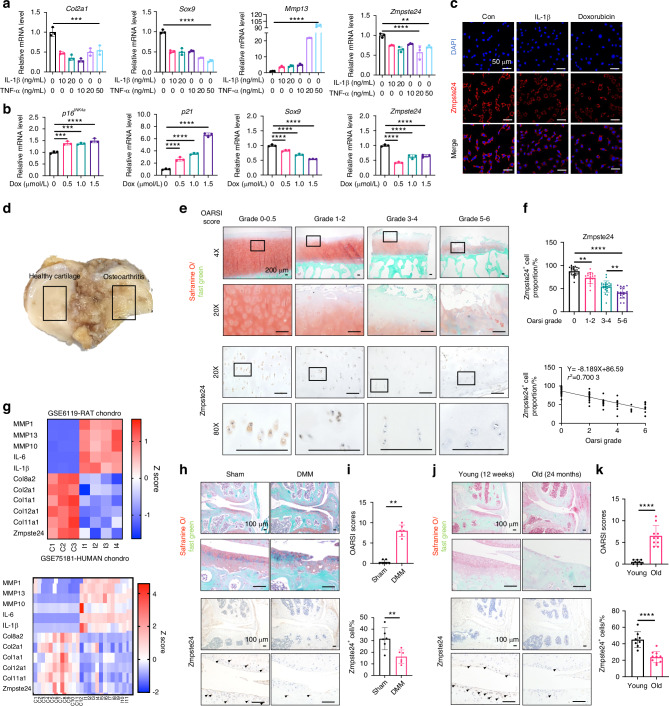
Reduced Zmpste24 expression in inflammatory and senescent microenvironments. **a**, **b** RT-qPCR of *Col2a1*, *Sox9*, *Mmp13*, and *Zmpste24* in ATDC5 cells treated with IL-1β, TNF-α, or doxorubicin. **c** Immunofluorescence staining of Zmpste24 after IL-1β and doxorubicin treatment. Scale bar: 50 μm. **d** Schematic of patient cartilage sample collection. **e** Safranin O and Zmpste24 immunohistochemical staining of cartilage samples with different severity. Scale bar: 200 μm. **f** Quantification of Zmpste24-positive cells and correlation with OARSI Grade. **g** Heatmap of Zmpste24 and cartilage metabolism-related genes in inflammatory GEO datasets. **h**, **i** Safranin O and Zmpste24 staining in mouse cartilage after DMM surgery. Scale bar: 100 μm. **j**, **k** Safranin O and Zmpste24 staining in aged (24 months) vs. young (12 weeks) mice. Scale bar: 100 μm. Data are shown as mean ± SD. *P < 0.05, **P < 0.01, ***P < 0.001, and ****P < 0.000 1

To further investigate the involvement of Zmpste24 in OA, its expression was examined in various OA models. Stimulation of ATDC5 chondrocytes with inflammatory cytokines IL-1β and TNF-α decreased Zmpste24 expression by about 50% (Fig. 3a, c). Similarly, the simulation of cellular senescence with doxorubicin also led to decreased Zmpste24 expression by 50% (Fig. 3b, c). Furthermore, medial tibial plateau specimens from patients undergoing total knee arthroplasty were collected, with one side exhibiting severe wear and the other served as a control with relatively mild wear (Fig. 3d). Immunohistochemical staining of Zmpste24 also showed decreased expression by 20% to 50% depending on degeneration severity (Fig. 3e, f). Correlation analysis revealed an inverse relationship between Zmpste24 expression and the degree of cartilage wear with a r^2^ of 0.7 (Fig. 3f).

Additionally, the gene expression profiles of rat and human chondrocytes in an inflammatory environment were collected to validate the decrease in Zmpste24 expression (Fig. 3g). Finally, post-traumatic arthritis and natural aging arthritis mouse models were established, and histological staining confirmed the successful establishment of OA (Fig. S8 and Fig. 3h–k). Immunohistochemical staining of Zmpste24 demonstrated a significant decrease in its expression in surgical and natural ageing arthritis models by 50% (Fig. 3h–k).

In conclusion, the expression of Zmpste24 decreases in inflammatory and aging microenvironments, indicating its involvement in arthritis and ageing.

### Lopinavir’s effects on chondrocyte degeneration and senescence were dependent on Zmpste24

After revealing the decreased expression of Zmpste24 in an inflammatory microenvironment, the effect of Zmpste24 knockdown on cartilage was further investigated. First, small interfering RNA (siRNA) was used to knock down Zmpste24 (Fig. S9a). It was found that Zmpste24 knockdown led to a decreased expression of Col2a1 and Sox9 and an increased expression of p16^INK4a^, p21, p53, and Mmp13 (Fig. S9b, c). Additionally, Zmpste24 global knockout mice were found to have a significantly smaller body size and weight than wild-type mice (Fig. S10a, b). Primary chondrocytes extracted from *Zmpste24*^*-/-*^ mice showed limited proliferation (Fig. S10c). Analysis of subchondral bone density revealed a significant decrease in mice lacking Zmpste24 by 40% in BV/TV (Fig. S10d, e), and knee joint cartilage staining demonstrated deteriorated cartilage degeneration in *Zmpste24*^*-/-*^ mice, with a significant reduction in the area and proportion of safranin O-positive chondrocytes (Fig. S10f, g).

**Fig. 4:**
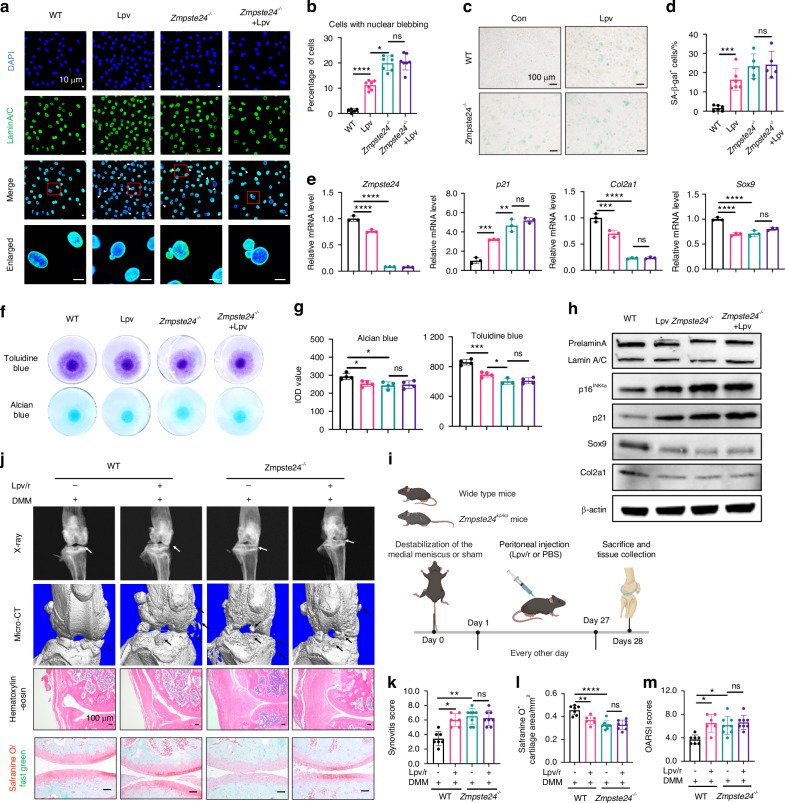
The effect of lopinavir on cartilage aging and degeneration depends on Zmpste24 inhibition. **a** Representative images of Lamin A immunofluorescence staining in primary chondrocytes from wild-type mice and *Zmpste24*^*-/-*^ mice after lopinavir treatment. Scale bar: 10 μm. **b** Quantitative analysis of nuclear blebbing cells. **c**, **d** SA-β-gal staining and quantitative analysis in primary chondrocytes from wild-type mice and *Zmpste24*^*-/-*^ mice after lopinavir treatment. Scale bar: 100 μm. **e** RT-qPCR experiment to detect the mRNA expression levels of *Zmpste24*, *Col2a1*, *Sox9*, and *p21* in mouse primary cells after lopinavir treatment. **f**, **g** Toluidine Blue and Alcian Blue staining of micromass of different primary chondrocytes after lopinavir treatment, and quantitative analysis. **h** Western Blot analysis of Col2a1, Sox9, Lamin A, p16^INK4a^, and p21 protein expression in different primary chondrocytes after lopinavir treatment. **i** Flowchart of the animal experiment. **j** Representative images of X-ray, micro-CT, HE staining, and Safranine O staining in wild-type mice and *Zmpste24*^*-/-*^ mice after further intraperitoneal injection of lopinavir/ritonavir following DMM surgery. Scale bar: 100 μm. *n* = 6, 7 or 8 per group. **k**–**m** Quantitative analysis of synovial inflammation score, Safranine O-positive cartilage area, and OARSI score. Data are shown as mean ± SD. *P < 0.05, **P < 0.01, ***P < 0.001, and ****P < 0.000 1

Next, we investigated whether lopinavir’s effects on cartilage degeneration depended on Zmpste24 inhibition. Primary chondrocytes from wild-type and *Zmpste24*^*-/-*^ mice were treated with lopinavir. Results showed that in *Zmpste24*^*-/-*^ chondrocytes, prelamin A band replaced the Lamin A band. Similar to the effects observed in cells treated with lopinavir, *Zmpste24*^*-/-*^ cells exhibited more cells with nuclear membrane blebbing and a 10-fold increased number of senescent cells (Fig. 4a–d). In addition, Zmpste24 knockout impaired cartilage matrix formation with a 15% decrease in alcian blue IOD value and 40% decrease in toluidine blue IOD value. Meanwhile, *Zmpste24*^*-/-*^ chondrocytes showed a 40% to 50% decrease in Sox9, Col2a1 mRNA and protein expression, and an increased expression of p16^INK4a^, p21 (Fig. 4e–h). In particular, these changes were not exacerbated by the additional treatment with lopinavir (Fig. 4a–h). For in vivo experiments, lopinavir/ritonavir was injected into wild-type and *Zmpste24*^*-/-*^ mice and the experimental design is shown in Fig. S11a. Staining showed that intraperitoneal injection of lopinavir/ritonavir under physiological conditions did not worsen cartilage degeneration or synovial inflammation in *Zmpste24*^*-/-*^ mice (Fig. S11b–g).

Furthermore, lopinavir/ritonavir was injected into the DMM-treated mice (Fig. 4i). X-ray and micro-CT results showed that lopinavir significantly increased joint space damage and osteophyte formation in wild-type mice, while in *Zmpste24*^*-/-*^ mice, lopinavir did not exacerbate osteophyte formation. Similar results were observed with haematoxylin and eosin (HE) and safranin O staining (Fig. 4j–m). Overall, these experiments indicate that the effects of lopinavir on cartilage degeneration depend on the inhibition of Zmpste24.

### Zmpste24 deficiency activated Usp7/Mdm2/p53 pathway by suppressing Usp7-mediated Mdm2 deubiquitination

**Fig. 5:**
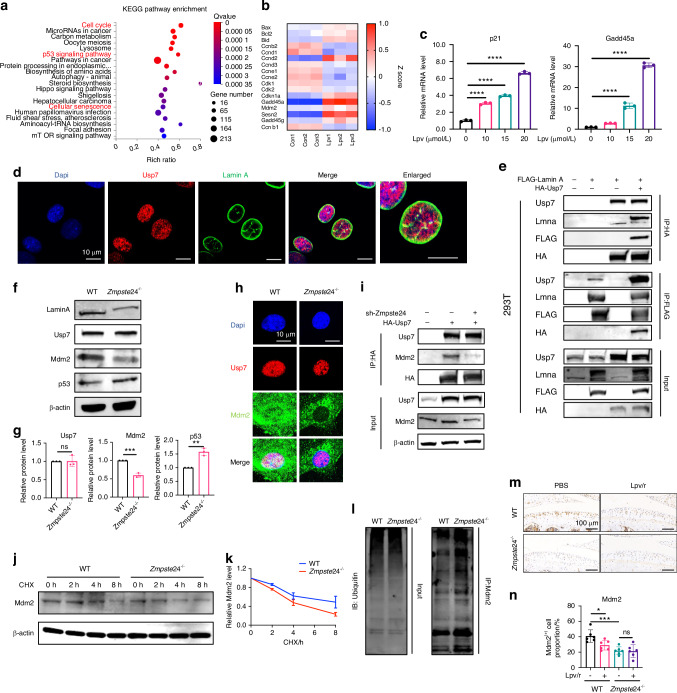
Zmpste24 deficiency induces senescence via Usp7/Mdm2/p53 pathway activation. **a** KEGG enrichment of differentially expressed genes after Zmpste24 inhibition by lopinavir. **b** Heatmap of p53 pathway-related gene expression. **c** RT-qPCR of *p21* and *Gadd45a* after lopinavir treatment. **d** Immunofluorescence co-localization of Usp7 and Lamin A. Scale bar: 10 μm. **e** Co-immunoprecipitation of Lamin A and Usp7. **f**, **g** Western blot and quantification of Lamin A, Usp7, Mdm2, and p53 in wild-type and *Zmpste24*^*-/-*^ chondrocytes. **h** Immunofluorescence co-localization of Usp7 and Mdm2. Scale bar: 10 μm. **i** Immunoprecipitation and Western blot of Mdm2 binding to Usp7 after Zmpste24 knockdown. **j**, **k** Mdm2 degradation rate after cycloheximide treatment. **l** Mdm2 ubiquitination levels in primary chondrocytes. **m**, **n** Mdm2 immunohistochemical staining and quantification. Scale bar: 100 μm. n = 6/group. Data are shown as mean ± SD. *P < 0.05, **P < 0.01, ***P < 0.001, and ****P < 0.000 1

Next, we explored the specific molecular mechanisms underlying cellular senescence caused by Zmpste24 deficiency. Transcriptomic data from cells treated with lopinavir indicated the activation of the p53 pathway (Fig. S12 and Fig. 5a, b). The activation of downstream factors in the p53 pathway under Zmpste24 inhibition was validated (Fig. 5c). Usp7 and Mdm2 are classical upstream regulators of the p53 pathway. Usp7 deubiquitinates Mdm2, thereby reducing its degradation, whereas Mdm2 inhibits p53 activation and accelerates p53 degradation. Immunofluorescence demonstrated the co-localization of Lamin A and Usp7 (Fig. 5d). An interaction between Lamin A and Usp7 was observed in 293T cells by co-immunoprecipitation (Fig. 5e), and similar results were obtained in ATDC5 cells (Fig. S13a). However, *Zmpste24*^*-/-*^ cells showed no change in Usp7 protein expression, whereas the expression of downstream Mdm2 decreased (Fig. 5f, g). Immunofluorescence experiments showed a significant reduction in the protein levels of Mdm2 combined with Usp7 after Zmpste24 knockout and a significant decrease in the interaction between Usp7 and Mdm2 after Zmpste24 knockdown (Fig. 5h, i and Fig. S13b). Degradation tests and ubiquitination level detection on Mdm2 revealed that in *Zmpste24*^*-/-*^ cells, the degradation rate of Mdm2 after cycloheximide-induced protein synthesis inhibition was significantly higher than in wild-type cells (Fig. 5j, k). Additionally, the ubiquitination level of Mdm2 was elevated in *Zmpste24*^*-/-*^ cells (Fig. 5l). These results indicate that Zmpste24 knockout enhances the ubiquitination of Mdm2 and accelerates its degradation through Lamin A-mediated modulation of the interaction between Usp7 and Mdm2, thereby facilitating the activation of the p53 pathway. Furthermore, Mdm2 expression was reduced in both wild-type mice and Zmpste24 knockout mice treated with lopinavir; however, lopinavir injection did not further decrease Mdm2 expression in *Zmpste24*^*-/-*^ mice (Fig. 5m, n). Subsequently, Mdm2 overexpression of chondrocytes showed that, under physiological conditions, cartilage matrix synthesis, cellular senescence, and the expression of senescence-related genes were not affected. However, in lopinavir-treated cells, Mdm2 overexpression significantly rescued the cartilage degeneration and senescence caused by lopinavir (Fig. S14). Overall, after inhibiting Zmpste24, lopinavir weakened the deubiquitylation function of Usp7 on Mdm2, leading to accelerated degradation of Mdm2 and activation of the p53 pathway, resulting in accelerated chondrocyte degeneration.

### Zmpste24 overexpression alleviated OA development

**Fig. 6:**
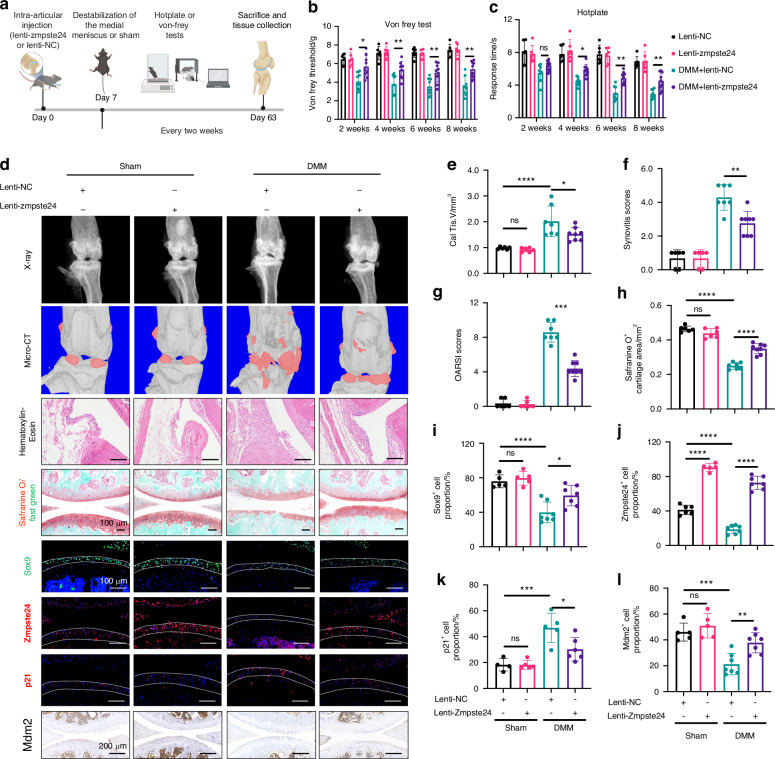
Overexpression of Zmpste24 in cartilage can delay the progression of osteoarthritis. **a** Flowchart of the animal experiment. **b** Von Frey pain threshold testing at different time points. **c** Hot plate test response time in mice at different time points. **d** Representative images of X-ray, micro-CT, HE staining, Safranine O/Fast Green staining, immunofluorescence staining for Sox9, p21, Zmpste24, and immunohistochemical staining for Mdm2 in different groups of mice. Scale bar: 100 μm. **e**–**i** Quantitative analysis of calcified tissue volume (**e**), synovitis score (**f**), OARSI score (**g**), Safranine O-positive cartilage area (**h**), Sox9 (**i**), Zmpste24 (**j**), p21 (**k**), and Mdm2 (**l**) positive cell ratios in different groups of mice. Data are shown as mean ± SD. *P < 0.05, **P < 0.01, ***P < 0.001, and ****P < 0.000 1

**Fig. 7:**
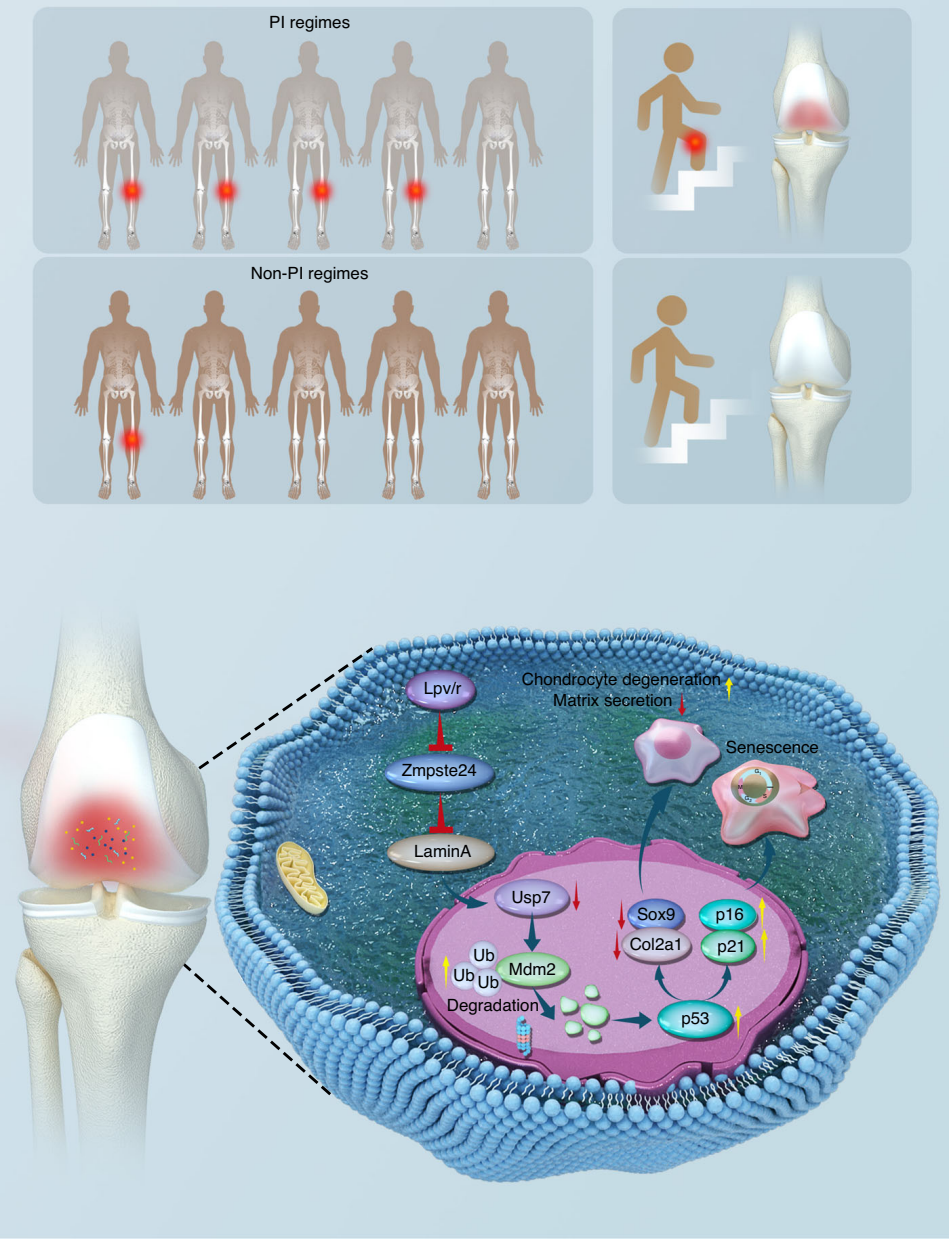
Schematic diagram of this study. Patients in the PI group had lower KOOS scores and a higher incidence of radiological knee OA compared to the non-PI group. Mechanistically, lopinavir-induced Zmpste24 inhibition compromised nuclear membrane stability, reducing the interaction between the nuclear membrane-binding protein Usp7 and Mdm2. This disruption activated the Usp7/Mdm2/p53 pathway, accelerating cellular senescence

After elucidating the role of Zmpste24 in cartilage senescence, we explored its therapeutic potential for OA alleviation. The experimental design is shown in Fig. 6a. Zmpste24 overexpression lentivirus was injected into the knee joint cavities of mice, and the mice’s perception of pain was assessed using the Von Frey and hot plate tests. The results showed that mice in the DMM group were more sensitive to thermal and mechanical pain, whereas Zmpste24 overexpression significantly reduced this sensitivity threshold by 40% (Fig. 6b, c). Radiological results indicated that Zmpste24 overexpression reduced osteophyte formation by 25% (Fig. 6d, e), whereas histological staining suggested that it relieved synovitis and cartilage erosion (Fig. 6d, f–h). Zmpste24 overexpression rescued the DMM surgery-induced decrease in Sox9 expression and increase in p21 expression. Mdm2 expression decreased after DMM surgery, whereas Zmpste24 overexpression reversed this trend (Fig. 6d, i–l). Overall, Zmpste24 overexpression effectively slowed OA progression. A schematic diagram of this study is shown in Fig. 7.

## Discussion

In this study, we established a retrospective cohort and found that an antiviral scheme involving PIs (including lopinavir) was likely to accelerate the occurrence of early OA. Furthermore, we proved that lopinavir could promote chondrocyte senescence and degeneration in vitro and in vivo after screening all available anti-HIV drugs. Finally, we demonstrated that the downregulation of Zmpste24 and activation of the Usp7/Mdm2/p53 pathway bridge the gap between lopinavir use and OA progression and that overexpression of Zmpste24 could rescue cartilage degeneration in OA.

With a longer life expectancy for PLWH, increased focus has been placed on morbidity and mortality from non-AIDS chronic age-related comorbidities. Our study is the first to establish a robust association between the use of PIs (especially lopinavir) and knee OA progression. This retrospective cohort study demonstrated a relationship between PI use and early OA symptoms. After screening all the available antiretroviral drugs, we found that PIs, particularly lopinavir, inhibit matrix anabolism. Furthermore, we validated the effect of lopinavir on knee OA progression both in vitro and in vivo by downregulating Zmpste24 expression. Zmpste24 deficiency inhibited Lamin A maturation and activated the Usp7/Mdm2/p53 pathway by increasing Mdm2 ubiquitination and degradation. Stabilization of the nuclear membrane by Zmpste24 overexpression prevents OA progression.

The diagnosis of early-stage knee OA is typically based on pain and functional limitations, which last for >1 week, interspersed with periods of little or no pain.^31^ Radiographic results, such as X-rays, are of limited value in early-stage OA diagnosis because joint space narrowing might not appear in patients with early OA.^32^ The latest report from the CRiteria for the Early Diagnosis of OA group proposed that a model based on factors obtained from questionnaires and physical examinations showed satisfactory predictive performance.^33^ Consistently, in our results, the number of patients with a KL score ≥2, the traditional criterion for radiological OA, was much smaller than that with a low functional score. PLWH older than 45 years were more susceptible to PI-induced adverse effects and experienced worse function in our cohort study than their younger counterparts, suggesting that the combined use of lopinavir/ritonavir has a smaller impact on OA symptoms in young people. For individuals over 45 years of age, the use of PIs would significantly raise the risk of early knee osteoarthritis progression. Our in vivo animal experiments consistently found that if mice were not subjected to DMM surgery, intraperitoneal injection of lopinavir/ritonavir did not result in cartilage erosion, leading to OA. However, lopinavir/ritonavir treatment significantly accelerated cartilage abrasion and senescence after DMM surgery. These results suggest that PIs may exert a mild influence on initial knee osteoarthritis, but significantly accelerates osteoarthritis development. Likewise, it accelerates cartilage aging and degeneration in inflammatory or pro-senescent microenvironments.

Zmpste24 knockout mice have been used as a progeroid mouse model in many studies; Suo et al. have shown that conditional knockout of Zmpste24 in chondrocytes promotes the occurrence and development of OA, and mice at 6 months of age suffer from severe cartilage abrasion.^34–36^ However, no studies have examined the expression of Zmpste24 in inflammatory and senescent microenvironments. Our study employed different models, including IL-1β and doxorubicin-stimulated chondrocytes, post-traumatic OA and natural aging mice, and tibial platform specimens from patients undergoing total knee replacement to explore the downregulation of Zmpste24 in chondrocytes under inflammatory and senescent conditions, which further indicates the potential role of Zmpste24 in cartilage degeneration and senescence. Previous studies have speculated that lopinavir promotes cellular senescence by inhibiting Zmpste24.^16^ Our study found, for the first time, that exposing Zmpste24-knockout cells to lopinavir does not further aggravate cartilage degeneration and senescence, demonstrating that the role of lopinavir in accelerating cartilage senescence is dependent on Zmpste24.

Many studies have explored the mechanisms of cellular senescence caused by the Zmpste24 knockout. These results suggest that the p53 pathway,^37^ Sirt1,^38^ histone methyltransferase EZH2, and downstream heterochromatin markers H3K27me3,^34^ Suv39h1, and H3K9me3,^39^ as well as casein kinase 2 (CK2),^40^ are involved in the aging process caused by Zmpste24 deficiency. However, the intermediate process by which Zmpste24 regulates p53 remains unclear. Zmpste24 is localized to the endoplasmic reticulum, where it plays a crucial role in the maturation of nuclear membrane protein Lamin A. As a result, the majority of studies suggest that senescence associated with Zmpste24 dysfunction is primarily due to impaired Lamin A maturation and the subsequent disruption of downstream signaling pathways.^38,40^ Our study first discovered the interaction of Lamin A and Usp7. Upon knockdown of Zmpste24, the expression level of Usp7 remains unchanged; however, the binding of Usp7 to Mdm2 is reduced. This results in an increased ubiquitination of Mdm2, which accelerates its degradation via proteasomes, thereby activating the p53 pathway. Mdm2 is an oncogene, and UBX0101 was developed to inhibit Mdm2 and target the clearance of senescent synoviocytes.^6^ However, a phase 2 clinical trial declared that the drug was ineffective.^41^ Our experiment demonstrates that overexpression of Mdm2 can reverse lopinavir-induced senescence of chondrocytes in vitro; however, it carries safety risks related to cancer formation. Therefore, we chose to overexpress Zmpste24 to stabilize the nuclear membrane, an effect that has been previously confirmed in earlier studies.^42^ The results showed that overexpression of Zmpste24 restored Mdm2 expression, rescued chondrocyte degeneration and senescence, and slowed the progression of OA.

In conclusion, through a retrospective cohort analysis, cellular functional assays, and in vivo animal studies, we demonstrated that PIs, particularly lopinavir, lead to the early onset of osteoarthritis symptoms by inhibiting Zmpste24. This study provides new insights into drug regime-related early OA in PLWH and unveils a new mechanism behind Zmpste24-related senescence. Based on our findings, PLWH at high risk for knee OA should be cautious about their regimens and choose other regimens when there are alternatives available.

## Materials and methods

### Clinical study

#### Patients

Our retrospective cohort study recruited 151 patients with HIV infection of more than 1 year in September 2023 from outpatient clinics at Shanghai Public Health Clinical Center. All participants who met the following inclusion criteria were included: (1) Patients’ age between 30 and 80 years old. (2) Patients who have been receiving at least 1 year of continuous HARRT therapy. Participants were excluded from the study if they ever had following diseases before medication: (1) Lower limb injury history; (2) Lower limb developmental abnormalities; (3) Lower limb deformities or history of lower limb fractures; (4) Pre-existing lower limb joint dysfunction; (5) Bilateral lower limb length discrepancy; (6) history of lower limb surgical infections; (7) History of intra-articular injections; rheumatoid arthritis or other inflammatory joint diseases; Paget’s disease; synovial chondromatosis; joint infection; osteochondroma; gout; osteopetrosis and other nervous system diseases affecting lower limb mobility such as spine degeneration or brain injury; (8) Inability to complete the questionnaire; (9) Inability to complete imaging examinations.

All patients were separated in two cohorts depending on whether they have received regimes containing lopinavir/ritonavir for more than 1 year. It is important to notice that lopinavir/ritonavir is the only available PI option in Shanghai.

The study was approved by the ethics committee of Shanghai Ninth People’s Hospital affiliated to Shanghai Jiao Tong University School of Medicine (SH9H-2022-T285-1). All patients gave informed consent.

#### Outcomes

Knee Injury and Osteoarthritis Outcome Score (KOOS score) was acquired through questionnaires. Radiological examinations (anterior and lateral radiographs of both knees) were done and evaluated by two experienced joint surgeons (ZZ and HL) who were blinded to the grouping of patients in Shanghai Ninth People’s Hospital. Knee radiographs were graded based on the Kellgren-Lawrence (KL) grading system^43^ from 0 to 4 (0, no OA; 1, doubtful OA; 2, minimal OA; 3, moderate OA; 4, severe OA). Physical examinations were done by experienced joint surgeons (ZZ and HL) and positive clinical signs were defined as presence of knee joint space tenderness, patellofemoral joint tenderness, or patellofemoral joint grinding pain, either individually or in combination. KOOS score is a knee specific instrument to assess patients’ opinions about their knees^44^ and it consists of 5 subscales: including Pain, other Symptoms, Activities of Daily Living (ADL), Sport and Reaction Function (Sport/Rec) and knee-related Quality of Life (QOL). The score ranges from 0 to 100, with 0 representing worse problems and 100 representing no problems for each subscale. A total score has not been validated and is not recommended.

#### **Covariates**

Baseline characteristics including demographic characteristics (i.e., age, sex, and postmenopausal status), HIV features (i.e., durations of HIV infection, CD4 level and viral load) and metabolic features (i.e., BMI, triglycerides, total cholesterol, HDL-cho, LDL-cho and glycaemia) were assessed using the nearest available data prior to the index date.

### Basic study

#### Human cartilage specimen collection

Cartilage samples were acquired with patients’ informed consent with ethnic approval from the ethics committee of the Shanghai Ninth People’s Hospital affiliated to Shanghai Jiao Tong University School of Medicine (SH9H-2021-T401-4). Human tibial plateaus were collected from patients undergoing TKA (Total knee arthroplasty) (*n* = 41; aged 70.6 ± 8.0 years; 10 males and 31 females). we drilled an osteochondral plug with an area of 0.5 cm × 0.5 cm in center of medial and lateral part of tibial plateau. The removed plugs were fixed in 4% paraformaldehyde for further histological assessment (Safranine O/Fast Green staining) and Zmpste24 immunohistochemistry.

#### Animals

Zmpste24 globally knockout mice (*Zmpste24*^*-/-*^) were bought from GemPharmaTech. Routine genotyping of mice tail DNA was performed according to the instruction of Jackson Laboratory with PCR kit (Takara, Japan). Homozygous mice (*Zmpste24*^*-/-*^) used in this study were all bred from heterozygous mice (*Zmpste24*^*+/-*^). Wild-type C57BL/6J mice were purchased from the animal center of Shanghai Ninth People’s Hospital. All mice were of C57BL/6J background. All mice were provided with a standard diet and housed in specific pathogen free (SPF) cages at a temperature of 24°C and a humidity of 60%. All animal experiments were approved by ethics committee of the Shanghai Ninth People’s Hospital affiliated to Shanghai Jiaotong University School of Medicine (SH9H-2022-A858-1).

For mice receiving the destabilization of the medial meniscus (DMM) surgery, we transected the medial meniscotibial ligament to enable destabilization of the medial meniscus, which is in accordance with the protocol of previous study.^45^

For the senile OA model, we fed C57BL/6J mice normally and sacrificed them at 24 months old, with 3-month-old mice as controls. Both lower extremities were collected and fixed for further radiological and histological analysis.

#### Drug administration

The mice were administered intraperitoneally PBS (control), Lopinavir/Ritonavir (100/25 or 200/50 mg/kg) every other day beginning 1 week after DMM surgery. Lopinavir/Ritonavir combination was dissolved in corn oil and the drug concentration was in accordance with the study of Alonso et al.^36^ In our study, for the in vitro experiments, we only used lopinavir to stimulate the chondrocytes. However, for the in vivo experiments, we combined lopinavir with ritonavir in accordance with the component ratio for patients. It is important to note that ritonavir was not used for its antiviral activities but rather as a booster for other antiviral drugs. Therefore, we only used the combination of lopinavir and ritonavir in the in vivo experiments to simulate the situation when patients take these medications in clinical practice.

#### Hotplate pain assay

The mice were placed on the hotplate at 55 °C.^46,47^ Response time was recorded as the period between hindlimbs touching the hotplate and the occurrence of response behaviors such as paw shaking, paw licking, or jumping. The hotplate pain assay was performed every 2 weeks after DMM surgery, and at least three replicative response times were recorded for each mouse. The observers were blinded to the grouping of all tested mice.

#### Von Frey tests

Von Frey test was carried out to measure the pain threshold in hindlimbs of mice using an electronic Von Frey filament (Xinruan Technology, China). Each mouse was allowed to acclimate to the test chamber for 30 min before the test. A positive response was recorded if the animal exhibited any nociceptive behavior such as paw withdrawal, shaking, or licking. The threshold force exerted by the electronic filament was recorded, with at least three replicative forces measured for each mouse in each test.

#### Micro-CT

After a 2-day fixation period, knee joints obtained from *Zmpste24*^*-/-*^ mice were scanned using a high-resolution micro-CT scanner (Skyscan 1072, Belgium) with a pixel size of 9 μm, a 55 kVp source, and a 145 μAmp current. Osteophyte volume was quantified, and representative three-dimensional reconstructions of subchondral bone sections were generated using CT-Vox software (Bruker, Germany), as described in our previous study.^14^

#### Histologic analyses

Serial tissue sectioning of 5 μm thickness in a sagittal or coronal plane was performed and stained with Safranine O/Fast Green to evaluate cartilage degeneration and clefts. OARSI score was evaluated according to the standards of Sophocleous.^45^ The OARSI score for each mouse knee joint is obtained by adding the OARSI scores of the femur and tibia. Furthermore, sections were stained with Hematoxylin and eosin (HE) staining to evaluate synovial inflammation, and the synovitis score was calculated using Krenn’s principle.^48,49^

#### Compound library screening

A customized compound library was obtained from TopScience, which included 25 FDA-approved anti-HIV drugs from seven different categories (listed in Table S2). Each drug was added individually at a final concentration of 20 μmol/L to ATDC5 cells. Dimethylsulfoxide (DMSO) was used as a control. After 24 h of incubation, RNA was extracted from the cells for the detection of Col2a1 and Sox9 expression using RT-qPCR.

#### Co-immunoprecipitation (Co-IP)

For Co-IP, whole protein extracts were lysed in 1 mL of cell lysis buffer for IP (P0013, Beyotime, China) containing 1% protease inhibitor cocktail at 4 °C for 30 min. Anti-Flag or Anti-HA immunomagnetic beads (Bimake, China) were added to the proteins and thoroughly mixed for 1 h at room temperature. The immunomagnetic beads were then collected using a magnet, washed three times with tris-buffered saline and Tween 20, and mixed with 1X loading buffer (Biosharp). The mixture was denatured at 99 °C for 15 min, followed by an immunoblotting procedure.

#### Cell culture and reagents

Murine chondrocytes were isolated from newborn C57BL/6J mice following a previously established protocol.^50^ Chondrocytes were cultured in Dulbecco’s Modified Eagle Medium/Nutrient Mixture F12 (DMEM/F12) supplemented with 10% fetal bovine serum (FBS) and 1% penicillin-streptomycin (Gibco, Thermo Fisher Scientific, Waltham, MA, United States).

The immortalized mouse chondrocyte cell line ATDC5 and HEK293T cells were obtained from the Cell Bank of the Chinese Academy of Sciences (Shanghai, China). ATDC5 cells were cultured in DMEM with 4.5 g glucose/L, 5% FBS, and 1% penicillin-streptomycin. Cells were incubated in a humid environment at 37 °C with 5% CO2. Lopinavir, ritonavir, and doxorubicin were purchased from MCE (New Jersey, United States) and dissolved in DMSO. The final concentration of DMSO in the cell culture medium was maintained below 0.1%. IL-1β protein was purchased from Genscript.

#### Plasmid and lentiviral transfections

All plasmids and lentiviruses used for cell transfection, including HA-Usp7, Flag-Lamin A, sh-Zmpste24, and Lenti-Mdm2, were purchased from Obio Biotechnology. For transfection of 293T or ATDC5 cells with these plasmids, 10 μg of the target plasmid was mixed with 15 μL of lipofectamine 3000 (Thermo Fisher Scientific) and 10 μL of P3000. The mixture was incubated for 15 min and then added to the cells for a 48-h transfection. Lentivirus was added to the medium with an optimal multiplicity of infection (MOI) of 20 to infect the cells. Puromycin was added 48 h after infection to select for a stable Mdm2 overexpression cell line.

#### 3D culture

For 3D culture, 2 × 10^5^ primary chondrocytes were centrifuged for 5 min at 1 500 r/min after which the pellet was cultured in chondrogenic medium (MUXMX-90041, Cyagen). Culture medium was replaced twice a week. After 21 days of chondrogenic 3D culture, the pellets were fixed in 4% PFA, dehydrated with gradient concentration of alcohol and embedded in SAKURA Tissue-Tek O.C.T. Compound. 5 μm sections were sliced with a Leica freezing microtome followed by Safranine O, Alcian Blue and Toluidine Blue staining.

#### Immunofluorescence (IF)

For cell IF staining, cells were washed with PBS for three times and fixed with 4% PFA. After 0.1% Triton-X permeabilization for 10 min and 5% BSA block for 30 min at room temperature, cells were incubated with primary antibodies, including anti-Lamin A (4777, 1:100, CST), anti-Usp7 (4833, 1:100, CST) and anti-Mdm2 (66511-1-lg, 1:100, proteintech) at 4 °C overnight. Cells were incubated with corresponding secondary antibody, including goat anti-rabbit IgG H&L (Alexa Fluor 488, 1:1 000, Abcam) and goat anti-mouse IgG H&L (Alexa Fluor 555, 1:1 000, Abcam) at room temperature for 1 h in the next day. Nucleus was stained with DAPI and representative images were taken with a high-resolution Leica confocal microscope. ImageJ software was utilized for quantitative analysis.

#### Quantitative real-time PCR (qRT-PCR)

Total RNA was isolated from cells using the Total RNA Extraction Kit (R6812-01HP, Omega Bio-tek Inc., Norcross, GA, United States). qRT-PCR was performed using an ABI 7500 Sequencing Detection System (Applied Biosystems, Foster City, CA, USA). The primer sequences used for the target genes are listed in Table S3.

#### Statistical analysis

Baseline characteristics and outcomes of included patients were presented as number (percentage) for categorical variables and mean (standard deviation) for continuous variables. χ2 test was used for categorical variables and two sample *t*-test was used for continuous variables in order to evaluate the differences between protease inhibitor and non-protease inhibitor patients.

Multivariable linear regression was used to determine the association between PI use and KOOS scores, including Pain, Symptom, DL, Sport/Rec, QOL, etc. Standardized coefficients (β) and their 95% confidence intervals were calculated. Multivariable logistic regression was used to clarify the association between PI use and radiological outcomes. Odds ratios (OR) and their 95% confidence intervals were calculated. Four models that adjusted for different variables were used to evaluate the stability of the results. Model 1 was not adjusted for any covariate. Model 2 was adjusted for age and sex. Model 3 was further adjusted for metabolic features including BMI, triglycerides, total cholesterol, HDL-cho, LDL-cho and glycaemia. Model 4 was adjusted for terms in model 3 and HIV features, including durations of HIV infection, CD4 level, and viral load (undetectable or not). All statistical analyses were performed using SPSS version 25.0 (SPSS Inc., Chicago, IL, USA). All P values for our clinical study are two-sided. A *P* < 0.05 indicated a statistical significance.

In basic studies, for data with a normal distribution, the unpaired Student’s t-test was used for comparisons between two groups. One-way ANOVA followed by Turkey’s post hoc tests were used for comparisons among three or more groups. The Mann-Whitney and Kruskal-Wallis tests were used for non-normally distributed data. Spearman correlation analysis was used to test the correlation between OARSI score and Zmpste24 expression level, and regression equations were obtained using simple linear regression methods. GraphPad Prism 8.0 (GraphPad Software Inc., San Diego, CA, USA) was used for designing and drawing statistical charts. Statistical significance was set at *P* < 0.05.

## Supplementary information

Supplementary materials

## Data Availability

All data needed to evaluate the conclusions in the paper are present in the paper and/or the Supplementary Materials.
