# Supplementary material for: AIDS patients suffer higher risk of advanced knee osteoarthritis progression due to lopinavir-induced Zmpste24 inhibition
Source: Bone Res. 2025 Jun 3;13:58. doi: 10.1038/s41413-025-00431-2 (PMC12134061; doi:10.1038/s41413-025-00431-2)
Supplement: Supplementary file 1 — Supplementary materials [file 41413_2025_431_MOESM1_ESM.docx]

**Supplemental Material**

**AIDS patients suffer higher risk of advanced knee osteoarthritis progression due to lopinavir-induced ZMPSTE24 inhibition**

**Authors: Keyu Kong^1, #^, Li Liu^2, #^, Renfang Zhang^2, #^, Yongyun Chang^1^, Yueming Shao^2^, Chen Zhao^1^, Hua Qiao^1^, Minghao Jin^1^, Xuzhuo Chen^3^, Wentao Shi^4^, Xinru Wu****^1^, Wenxuan Fan^1^, Yuehao Hu^1^, Kewei Rong^1^, Pu Zhang^1^, Baixing Li^1^, Jingwei Zhang^1^, Peixiang Ma^1^, Xiaoling Zhang^5, *^, Huiwu Li^1, *^, Zanjing Zhai^1, *^**

**Table of contents**

Tables S1 to S3

Figs. S1 to S14

**Supplementary Table 1. Basic characteristics of all included participants.**

|  | **Total (n=151)** | **Protease Inhibitor (n=78)** | **non-Protease Inhibitor (n=73)** | **p value** |
| --- | --- | --- | --- | --- |
| ***Demographic features*** |  |  |  |  |
| *Male gender, n (%)* | 141 (93.4) | 68 (87.2) | 73 (100) | 0.002 |
| *Age (years), mean (SD)* | 43.7 (10.8) | 43.3 (10.8) | 44.1 (10.8) | 0.657 |
| *Postmenopausal status (n=10), n(%)* | 2 (20) | 2 (20) | 0 (0) | 0.999 |
| ***Metabolic features*** |  |  |  |  |
| *BMI (kg/m^2^), mean (SD)* | 22.9 (2.8) | 22.6 (2.9) | 23.2 (2.7) | 0.178 |
| *Overweight (BMI ≥ 25), n (%)* | 30 (19.9) | 15 (19.2) | 15 (20.5) | 0.842 |
| *Triglycerides (mmol/L), mean (SD)* | 2.38 (1.90) | 2.73 (2.28) | 2.00 (1.30) | 0.018 |
| *Total cholesterol*  *(mmol/L), mean (SD)* | 4.67 (0.92) | 4.77 (0.93) | 4.56 (0.91) | 0.172 |
| *HDL-cho (mmol/L), mean (SD)* | 1.22 (0.26) | 1.21 (0.27) | 1.22 (0.26) | 0.891 |
| *LDL-cho (mmol/L), mean (SD)* | 2.77 (0.80) | 2.70 (0.79) | 2.83 (0.81) | 0.310 |
| *Glycaemia (mmol/L), mean (SD)* | 5.79 (1.55) | 5.74 (1.34) | 5.84 (1.71) | 0.697 |
| ***HIV features*** |  |  |  |  |
| *Duration of HIV infection (years), mean (SD)* | 8.3 (4.5) | 8.6 (4.9) | 8.0 (3.9) | 0.409 |
| *CD4 level (/mm3), mean (SD) I* | 584.3 (224.1) | 578.1 (224.5) | 591.0 (225.0) | 0.727 |
| *Undetectable usHIV viral load, n (%)* | 142 (94.0) | 72 (92.3) | 70 (95.9) | 0.497 |

BMI, body mass index; cho, cholesterol; LDL, low-density lipoprotein; HDL, high-density lipoprotein; usHIV, ultrasensitive HIV.

**Supplementary Table 2. All anti-HIV drugs and their categories in compound library.**

| **NTRIs** | **NNTRIs** | **PIs** | **INSTIs** | **FIs** | **CRA** | **Attachment inhibitor** | |
| --- | --- | --- | --- | --- | --- | --- | --- |
| **Lamivudine** **(3TC)** | **Nevirapine**  **(NVP)** | **Ritonavir**  **(RTV)** | **Raltegravir potassium**  **(RAL)** | **Enfuvirtide acetate**  **(T20)** | **Maraviroc**  **(MVC)** | | **Fostemsavir**  **(FTR)** |
| **Zidovudine**  **(AZT)** | **Etravirine**  **(ETR)** | **Lopinavir**  **(LPV)** | **Elvitegravir**  **(EVG)** |  |  | |  |
| **Tenofovir alafenamide**  **(TAF)** | **Rilpivirine**  **(RPV)** | **Atazanavir**  **(ATV)** | **Cabotegravir**  **(CAB)** |  |  | |  |
| **Tenofovir**  **(TDF)** | **Efavirenz**  **(EFV)** | **Darunavir**  **(DRV)** | **Dolutegravir**  **(ETG)** |  |  | |  |
| **Emtricitabine**  **(FTC)** | **Doravirine**  **(DOR)** | **Saquinavir**  **(SQV)** | **Bictegravir**  **(BIC)** |  |  | |  |
| **Abacavir**  **(ABC)** |  | **Fosamprenavir Calcium Salt**  **(FPV)** |  |  |  | |  |

NTRIs, Nucleoside reverse transcriptase inhibitors; NNTRIs, Non-nucleoside reverse transcriptase inhibitors; PIs, Protease inhibitors; INSTIs, Integrase strand transfer inhibitors; FIs, Fusion inhibitors; CRA, CCR5 antagonists.

**Supplementary Table 3. Primers used in the qRT-PCR assay.**

| Gene | Organisms | Forward (5′-3′) | Reverse (5′-3′) |
| --- | --- | --- | --- |
| Gapdh | Mus musculus | GGCAAGTTCAACGGCACAG | CGCCAGTAGACTCCACGACAT |
| Col2a1 | Mus musculus | GCTACACTCAAGTCACTGAACAACCA | TCAATCCAGTAGTCTCCGCTCTTCC |
| Sox9 | Mus musculus | CGTGGACATCGGTGAACTGAG | GGTGCTGCTGATGCCGTAAC |
| p16^INK4a^ | Mus musculus | GGTCACACGACTGGGCGATT | GCACCGTAGTTGAGCAGAAGAG |
| p21 | Mus musculus | GCCTGGTTCCTTGCCACTTCTT | ATTACGGTTGAGTCCTAACTGCCATC |
| p53 | Mus musculus | CTCCAGCTACCTGAAGACCAAGAAG | GCAGAGACCTGACAACTATCAACCTAT |
| Mmp3 | Mus musculus | CCCTGCAACCGTGAAGAAGA | GACAGCATCCACCCTTGAGT |
| Mmp13 | Mus musculus | GGAGCCCTGATGTTTCCCAT | GTCTTCATCGCCTGGACCATA |
| Mmp9 | Mus musculus | CGACGACGACGAGTTGTGGT | GTTGCCGTGCTCCGTGTAGA |
| Adamts4 | Mus musculus | GGTCCTGGGCCCTAGTTGTATTTA | AAGCAGGGCAGGTTGTACCTCATA |
| Aggrecan | Mus musculus | CCTGCTACTTCATCGACCCC | AGATGCTGTTGACTCGAACCT |
| Zmpste24 | Mus musculus | CTTGGTAGGCAGGCAGATGTTGAC | CGCAGCAGGACTCTCACTTAGGA |
| Zmpste24 | Homo Sapiens | TGGACGCTTTGTGGGAGATGC | TGCCGCTGTGCTAGGAAGGT |
| Gadd45a | Mus musculus | CTGCTGCGAGAACGACATCA | CCACTGATCCATGTAGCGACTT |
| Usp7 | Mus musculus | GCTGATGAGTAGGCTCGTCCAA | CCACTTCGTGAACAGGCTGCTA |
| Mdm2 | Mus musculus | AAGAGCCATGTGCTGAGGAGGA | GGTGTCCAGTCTTGCCGTGAAC |
| Lbr | Mus musculus | TCACCGCCTTGCTTATCCATCG | GGCAAGCAGCACAGGTCAGTAA |
| Sun1 | Mus musculus | TTCTCGGTTCGTGAGCAAGGAT | GCAGACACAATAGCCTCGGATG |
| Sun2 | Mus musculus | CGCTACAGTGAGGACAGGATTG | CTGAGGAGTGCCGTCTTGGT |
| Lmna | Mus musculus | GAGATGCTGAGGCGAGTGGATG | GCTTGGTCTCACGCAGTTCCT |
| Lmnb1 | Mus musculus | GTGGTCTGCTCTCGGTCCTTCT | ACCTCCATCCAGAGTGCGTACA |
| Lmnb2 | Mus musculus | CGTGACAAGTTCCGCAAGATG | AGATGGTGATCCGTGATGAAGG |


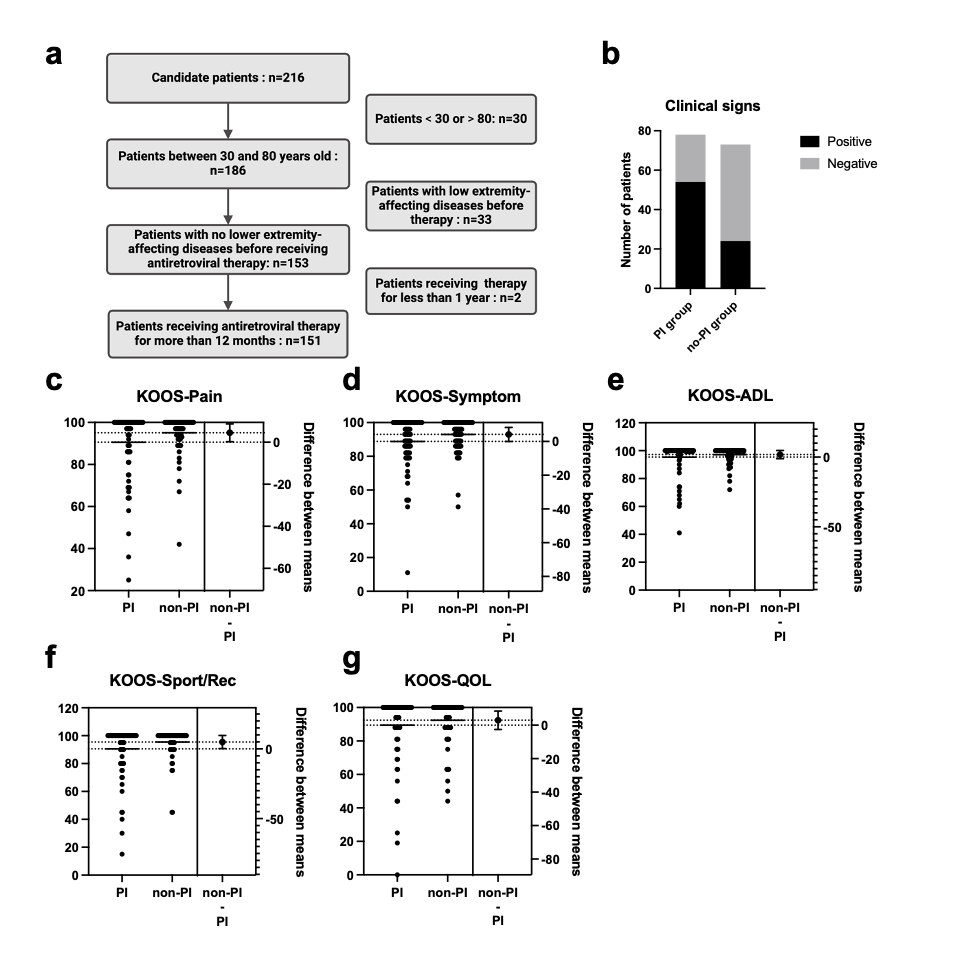


**Supplementary Figure 1: PI-containing regimens were related with higher incidence of positive clinical signs and fewer KOOS scores.**

(a) Selection process of included participant for comparison between PI group and non-PI group. (b) Bar chart of clinical sign incidence between two groups. (c-g) Individual value chart showing different subscores (KOOS pain (c), symptom (d), function in daily living (ADL) (e), function in sport and recreation (Sport/Rec) (f) and quality of life (QOL) (g)) of KOOS scores in PI group and non-PI group. PI, protease inhibitors; KOOS, knee Injury and Osteoarthritis Outcome Score.


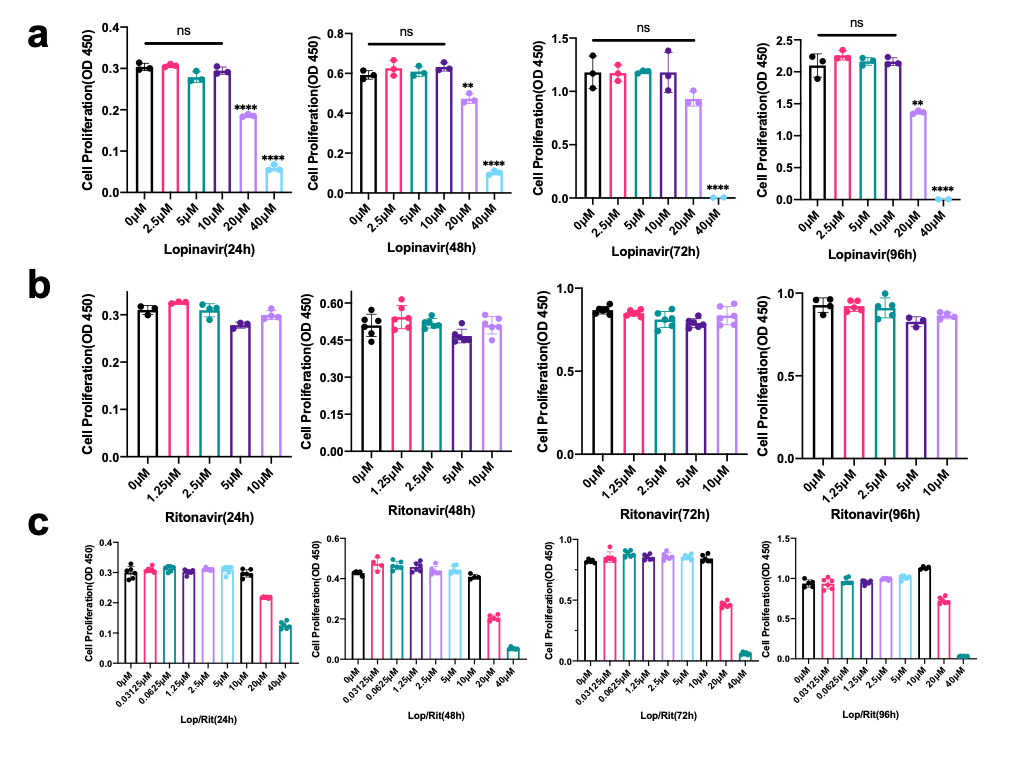


**Supplementary Figure 2: Lopinavir inhibition on cell proliferation.**

CCK-8 cell viability assay of ATDC5 chondrocytes treated with lopinavir (a), ritonavir (b) or lopinavir/ritonavir combination (c). Data are shown as mean ± SD. *P<0.05, **P<0.01, ***P<0.001, and ****P<0.0001.


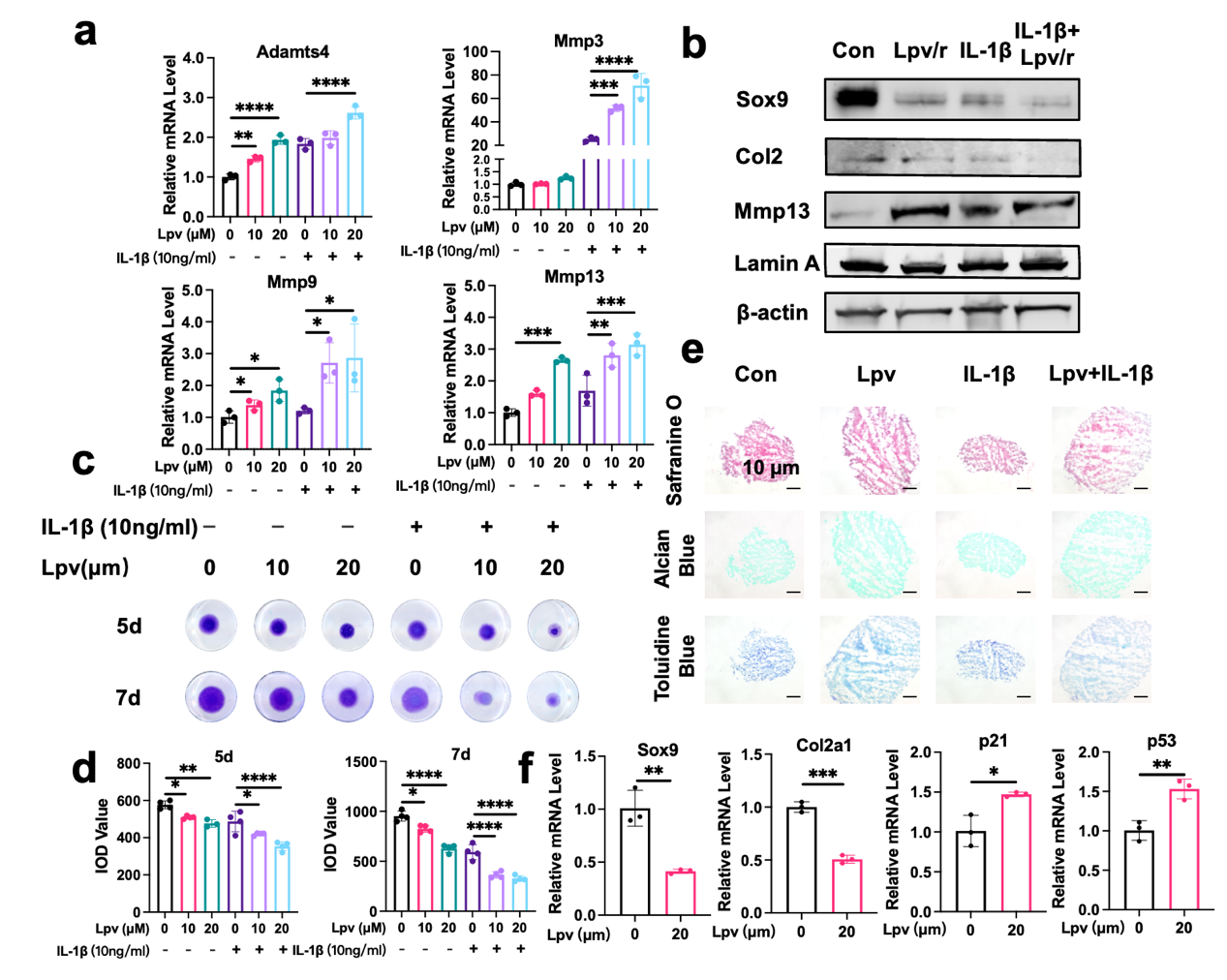


**Supplementary Figure 3: Effect of lopinavir/ritonavir on chondrocyte degeneration and senescence.**

(a) RT-qPCR analysis of Adamts4, Mmp3, Mmp9, and Mmp13 mRNA expression after treatment with lopinavir and IL-1β. (b) Effect of lopinavir/ritonavir on protein expression of Sox9, Col2a1, Mmp13 and Lamin A. (c, d) Toluidine Blue staining and quantitative analysis of micromass at different time points after treatment with lopinavir and IL-1β. (e) Representative images of Safranine O, Alcian Blue, and Toluidine Blue staining in 3D cultured cartilage pellets treated with lopinavir and IL-1β. Scale bar: 10 μm. (f) Gene expression of Col2a1, Sox9, p21 and p53 in primary murine chondrocytes treated with lopinavir. Data are shown as mean ± SD. *P<0.05, **P<0.01, ***P<0.001, and ****P<0.0001.

**
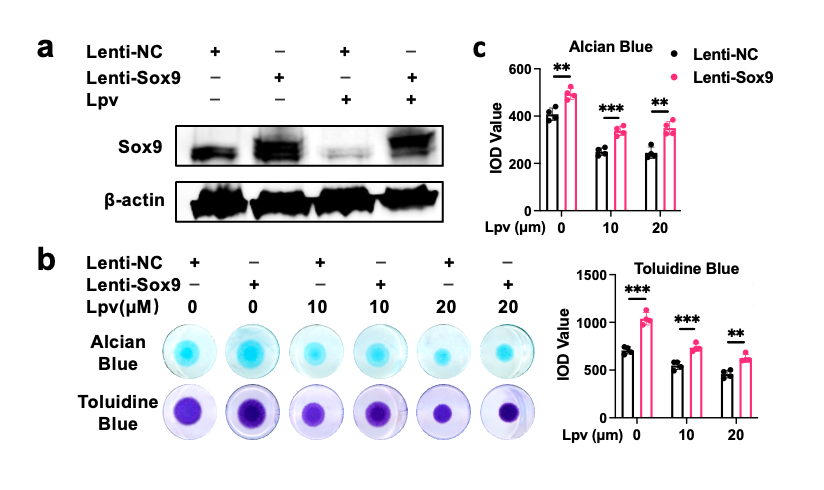
Supplementary Figure 4: Overexpression of Sox9 can rescue lopinavir-induced cartilage degeneration.**

(a) Western blot of Sox9 after lopinavir treatment and Sox9 overexpression. (b, c) Alcian Blue and Toluidine Blue staining with quantification in Sox9 overexpression and control groups. Data are shown as mean ± SD. *P<0.05, **P<0.01, ***P<0.001, and ****P<0.0001.


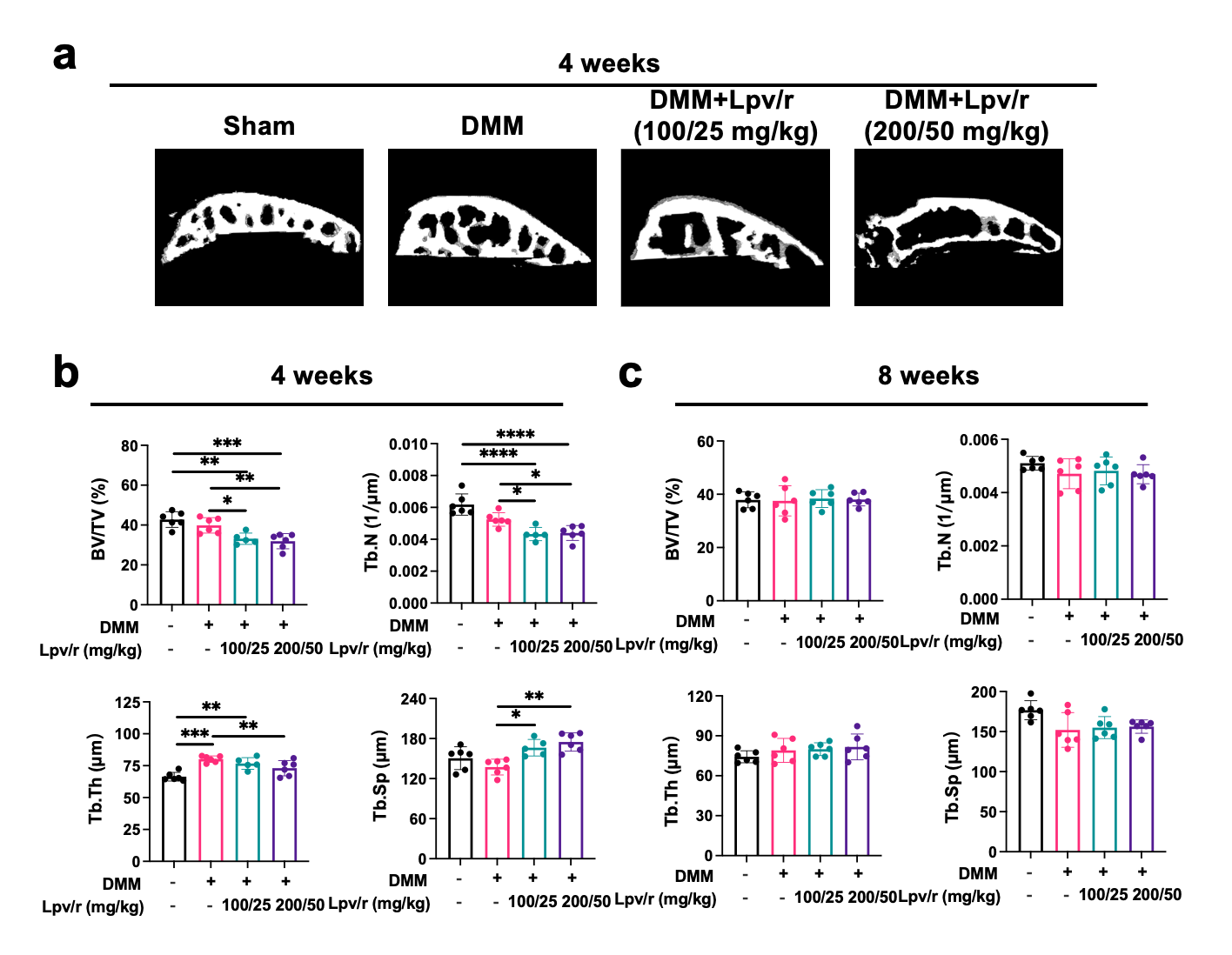


**Supplementary Figure 5: Subchondral bone analysis in mice at different time points after intraperitoneal injection of lopinavir following DMM surgery.**

(a) Representative images of subchondral bone cross-sections in the sagittal plane reconstructed by CT in mice four weeks after DMM surgery. n=5 or 6 per group. (b, c) Quantitative analysis of subchondral bone parameters including BV/TV, Tb.N, [Tb.Th](http://Tb.Th), and Tb.Sp in mice four weeks and eight weeks after surgery. Data are shown as mean ± SD. *P<0.05, **P<0.01, ***P<0.001, and ****P<0.0001.


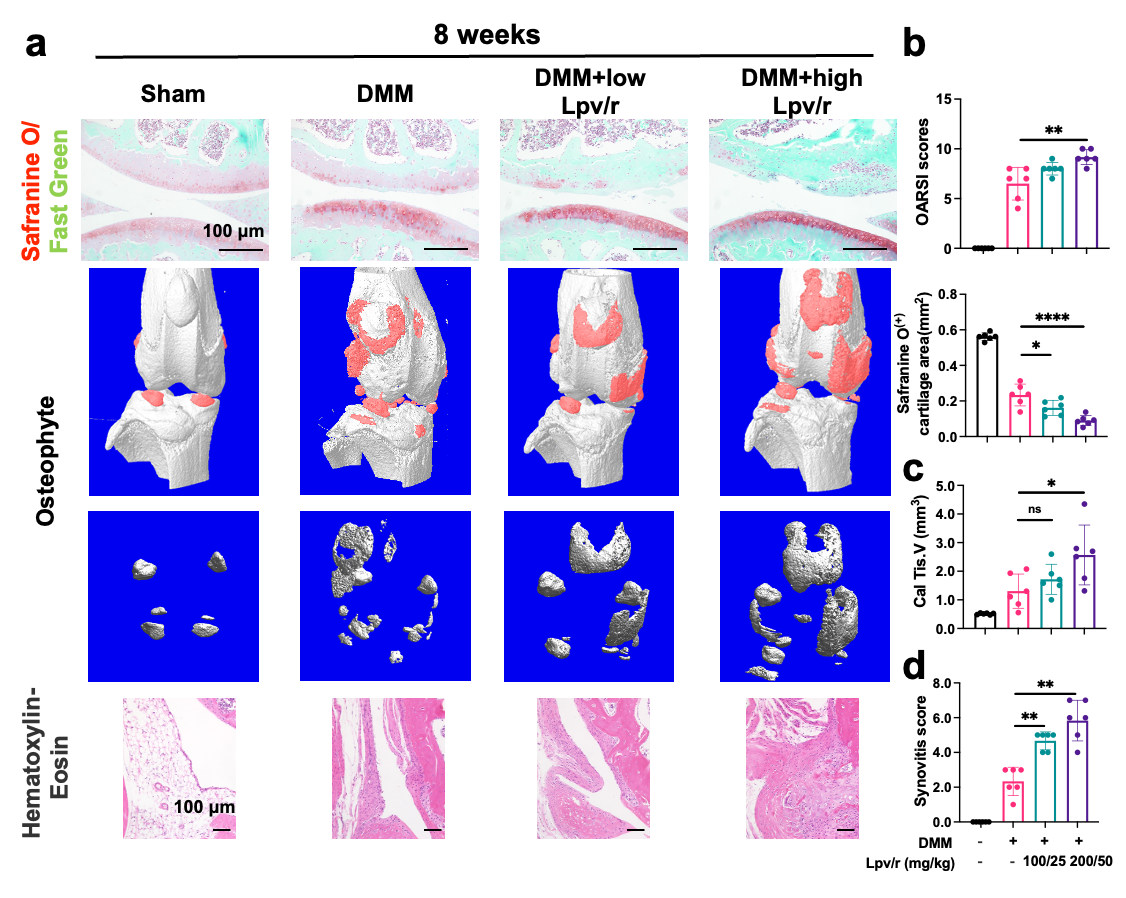


**Supplementary Figure 6: Histological analysis in mice at 8 weeks after intraperitoneal injection of lopinavir following DMM surgery.**

(a) Representative images of Safranine O/Fast Green staining, osteophyte 3D reconstruction, and H&E staining in the cartilage of mice at 8 weeks after DMM surgery. Scale bar: 100 μm. n=5 or 6 per group. (b-d) Quantitative analysis of OARSI score, Safranine O-positive cartilage area (b), calcified tissue volume (c), and synovitis score (d) in different groups of mice. Data are shown as mean ± SD. *P<0.05, **P<0.01, ***P<0.001, and ****P<0.0001.


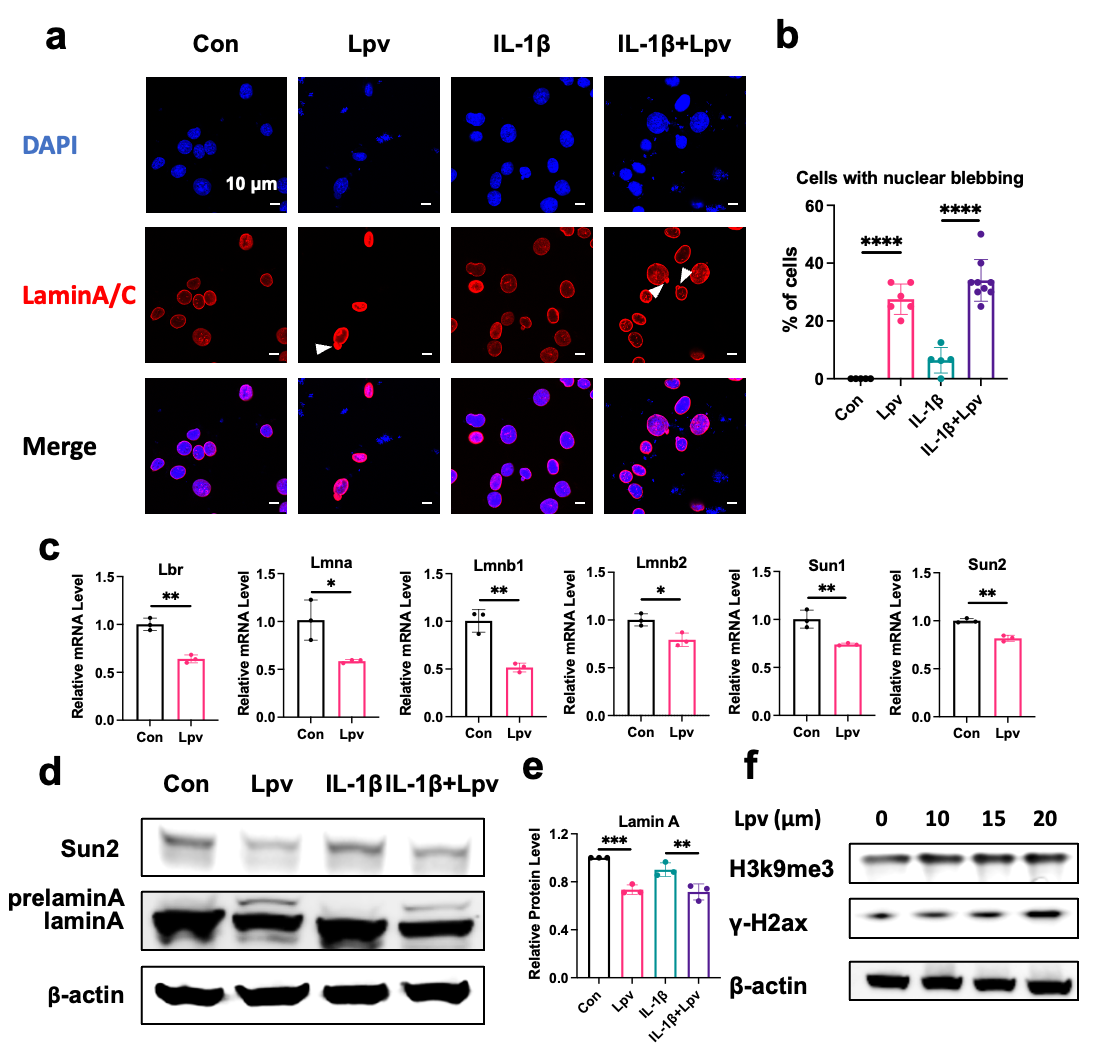


**Supplementary Figure 7: Lopinavir inhibits the function of Zmpste24.**

(a) Immunofluorescence staining of Lamin A in cells treated with IL-1β and lopinavir. Scale bar: 10 μm. (b) Quantitative analysis of nuclear extrusion. (c) qRT-PCR analysis of mRNA expression levels of nuclear envelope-related proteins Lbr, Lmna, Lmnb1, Lmnb2, Sun2, and Sun1 after lopinavir treatment. (d, e) Western Blot analysis and quantitative analysis of Sun2 and Lamin A protein expression after IL-1β and lopinavir treatment. (f) Western Blot analysis of H3K9me3 and γH2AX protein expression after treatment with different concentrations of lopinavir. Data are shown as mean ± SD. *P<0.05, **P<0.01, ***P<0.001, and ****P<0.0001.


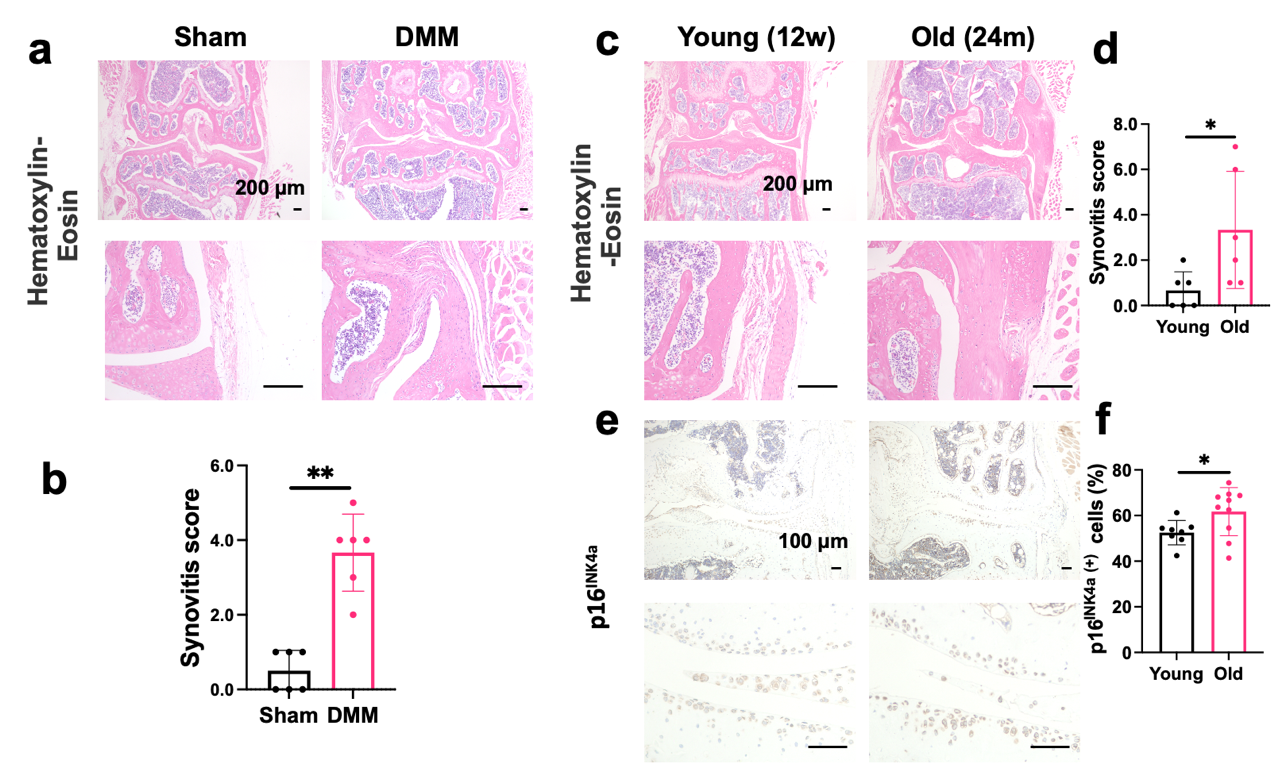


**Supplementary Figure 8: Validation of successful modeling in DMM and natural aging mouse models.**

(a, b) Representative H&E staining images and quantitative analysis of synovial inflammation in DMM group mice compared to Sham group mice. Scale bar: 200 μm. n=6 per group. (c, d) Representative H&E staining images and quantitative analysis of synovial inflammation in natural aging mice compared to young mice. Scale bar: 200 μm. n=8 or 10 per group. (e, f) Representative immunohistochemical staining images of p16^INK4a^ and quantitative analysis of positive cells in natural aging mice compared to young mice. Scale bar: 100 μm. Data are shown as mean ± SD. *P<0.05, **P<0.01, ***P<0.001, and ****P<0.0001.


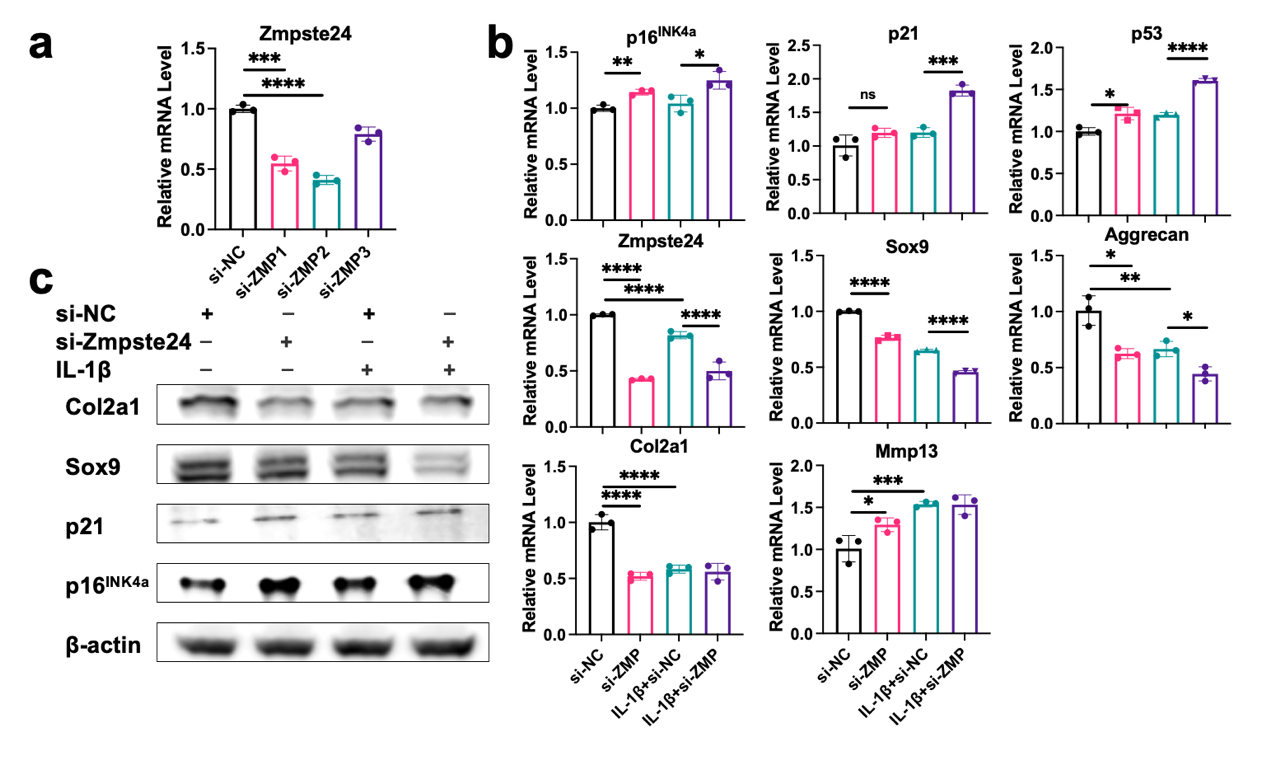


**Supplementary Figure 9: Small interfering RNA (siRNA) knockdown of Zmpste24 accelerates cellular senescence and degeneration.**

(a) Validation of mRNA efficiency of three siRNAs targeting Zmpste24. (b) RT-qPCR analysis of Zmpste24, Col2a1, Sox9, aggrecan, Mmp13, p16^INK4a^, p21, and p53 mRNA expression in cells after treatment with siRNA and IL-1β. (c) Western Blot analysis of Col2a1, Sox9, p16INK4a, and p21 protein expression in cells after treatment with siRNA and IL-1β. Data are shown as mean ± SD. *P<0.05, **P<0.01, ***P<0.001, and ****P<0.0001.


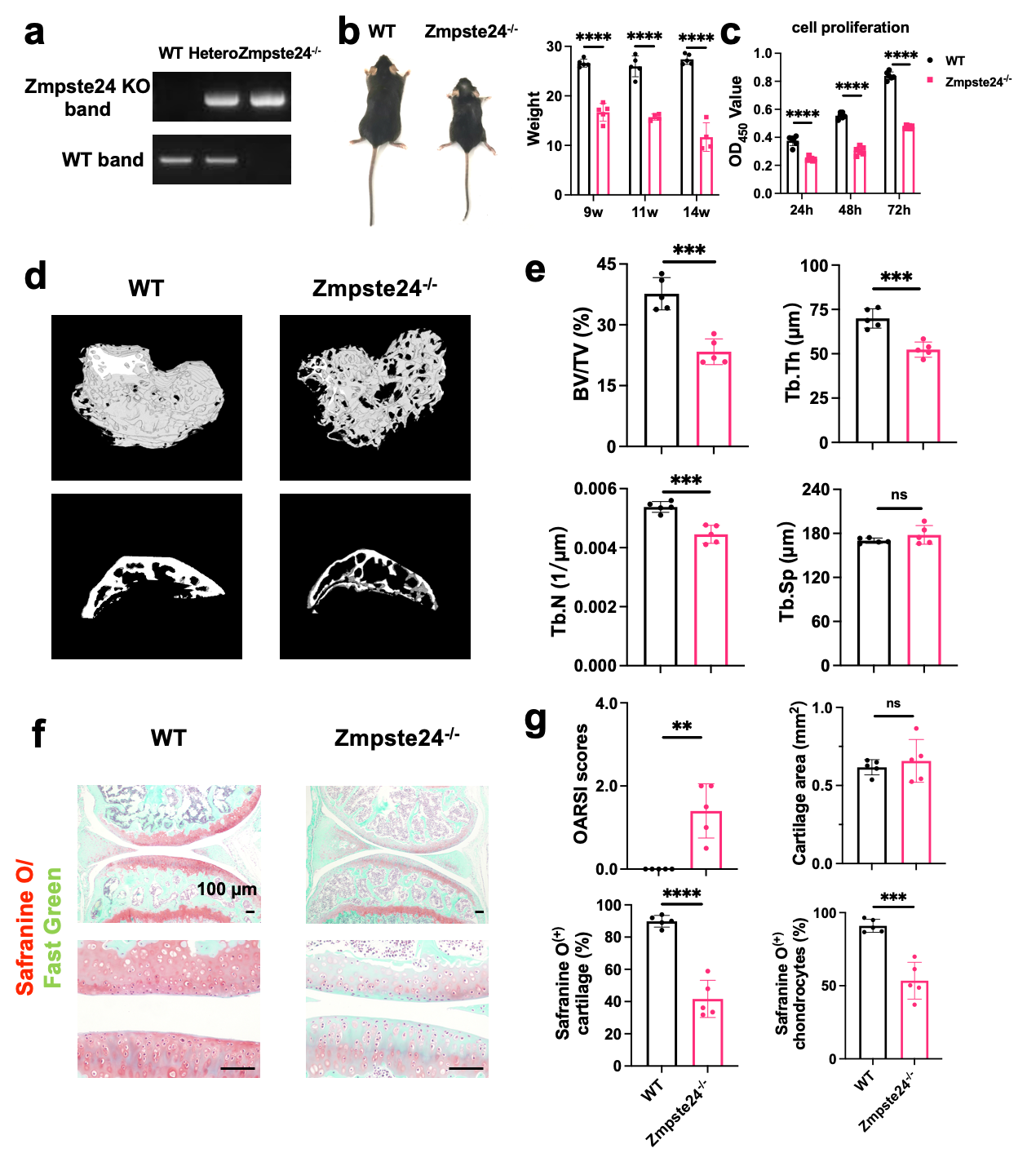


**Supplementary Figure 10: Phenotypic analysis of Zmpste24-/- mice.**

(a) Gene identification bands of Zmpste24-/- mice. (b) Measurement of body size and weight in Zmpste24-/- mice. (c) Cell proliferation assay of primary chondrocytes from wild-type mice and Zmpste24-/- mice. (d, e) Representative images of subchondral bone 3D reconstruction and quantitative analysis of subchondral bone density in different mice. n=5 per group. (f) Representative images of Safranine O staining in cartilage of wild-type mice and Zmpste24 -/- mice. Scale bar: 100 μm. n=5 per group. (g) Quantitative analysis of OARSI score, cartilage area, Safranine O-positive cartilage area, and proportion of Safranine O-positive chondrocytes in both groups of mice. n=5 per group. Data are shown as mean ± SD. *P<0.05, **P<0.01, ***P<0.001, and ****P<0.0001.


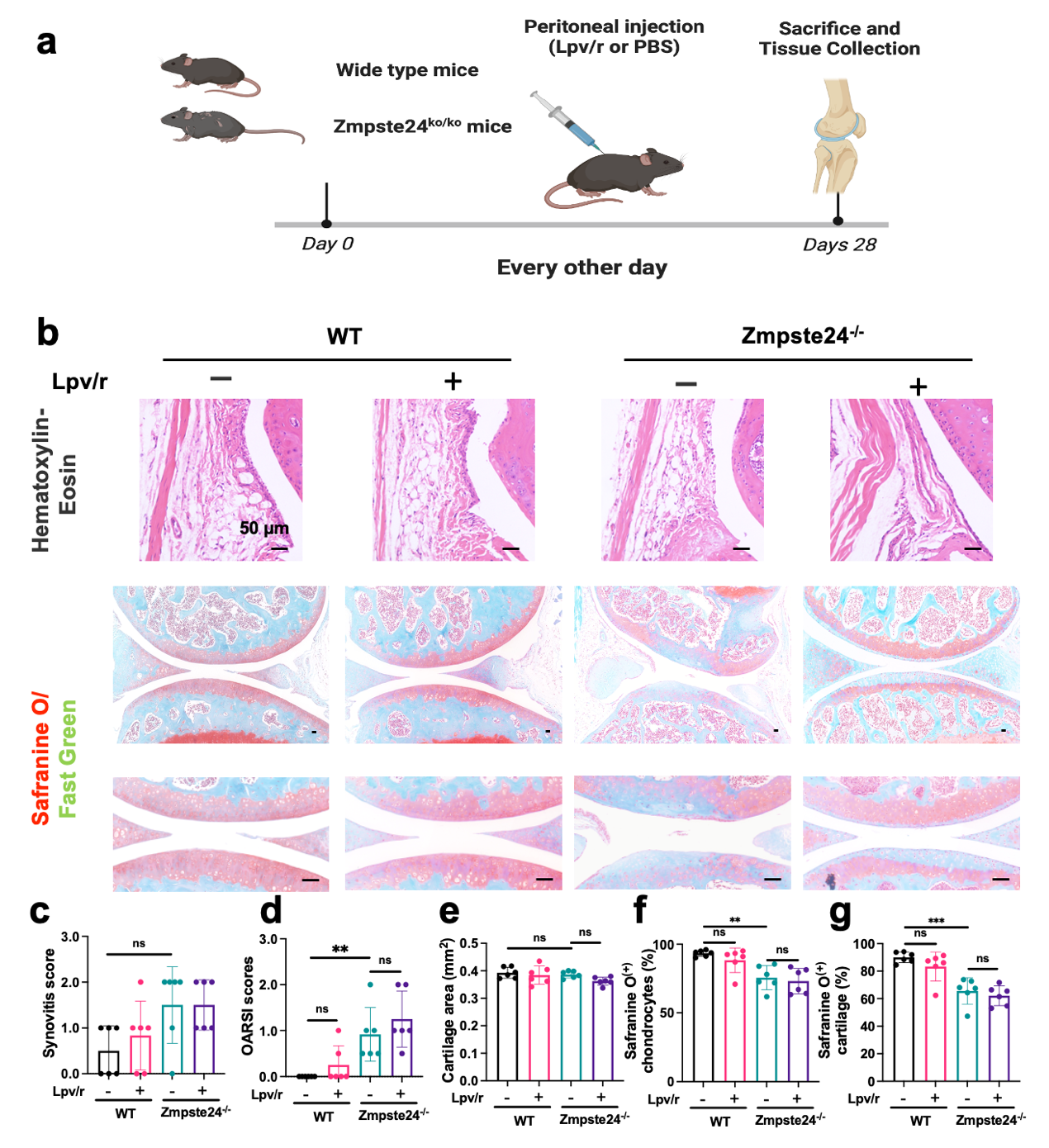


**Supplementary Figure 11: Effects of lopinavir on cartilage phenotype in wild-type and Zmpste24 knockout mice under physiological conditions.**

(a) Flowchart of the animal experiment. (b) Representative images of H&E staining and Safranine O staining in the knee joints of mice from different groups. Scale bar: 50 μm. n=6 per group. (c-g) Quantitative analysis of synovitis score (c), OARSI score (d), cartilage area (e), Safranine O-positive cartilage area (f), and proportion of Safranine O-positive chondrocytes (g) in different groups of mice. n=6 per group. Data are shown as mean ± SD. *P<0.05, **P<0.01, ***P<0.001, and ****P<0.0001.


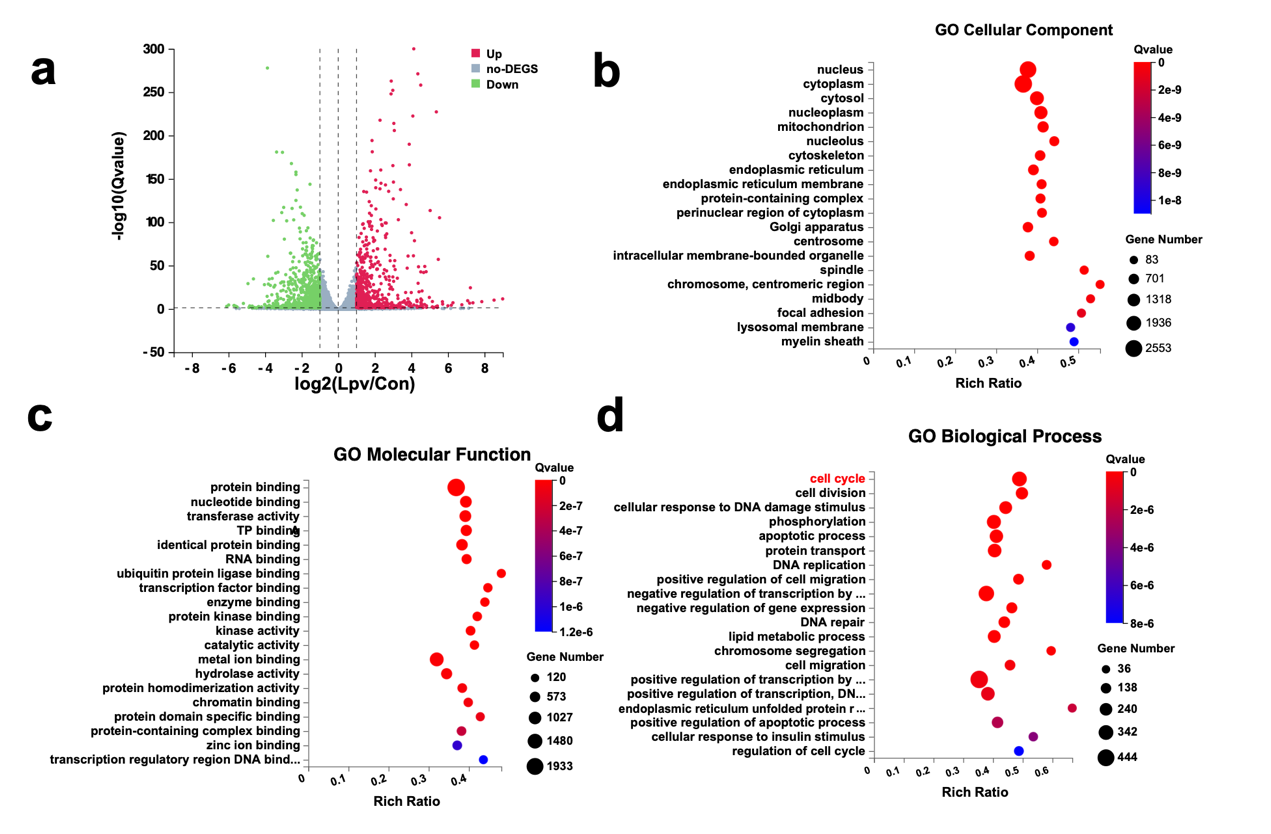


**Supplementary Figure 12: Volcano plot (a) and GO enrichment analysis (b-d) of differentially expressed genes after Zmpste24 inhibition.**


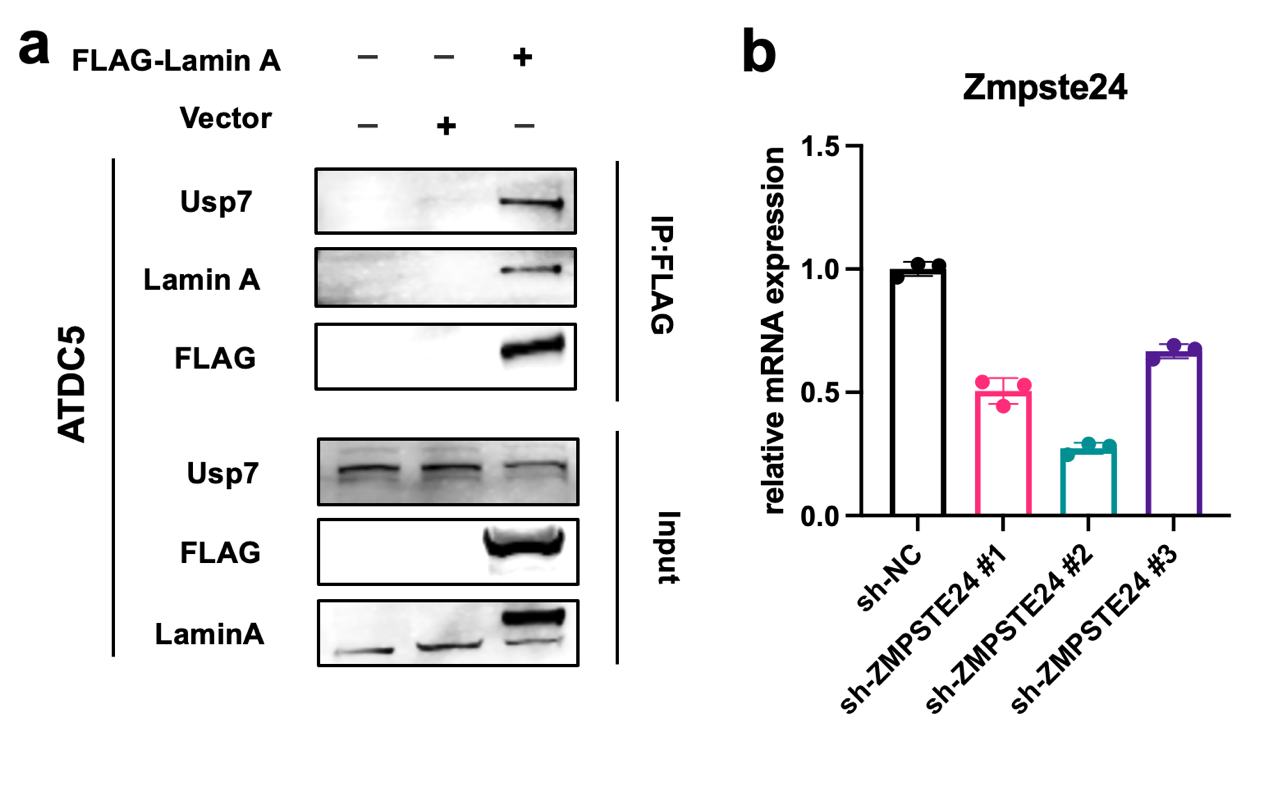


**Supplementary Figure 13: Mechanism of cellular senescence in Zmpste24 deficiency.**

(a) Co-immunoprecipitation experiment of exogenous Lamin A and Usp7 in ATDC5 cells. (b) Efficiency detection of three shZmpste24 on Zmpste24 knockdowns. Data are shown as mean ± SD. *P<0.05, **P<0.01, ***P<0.001, and ****P<0.0001.

**
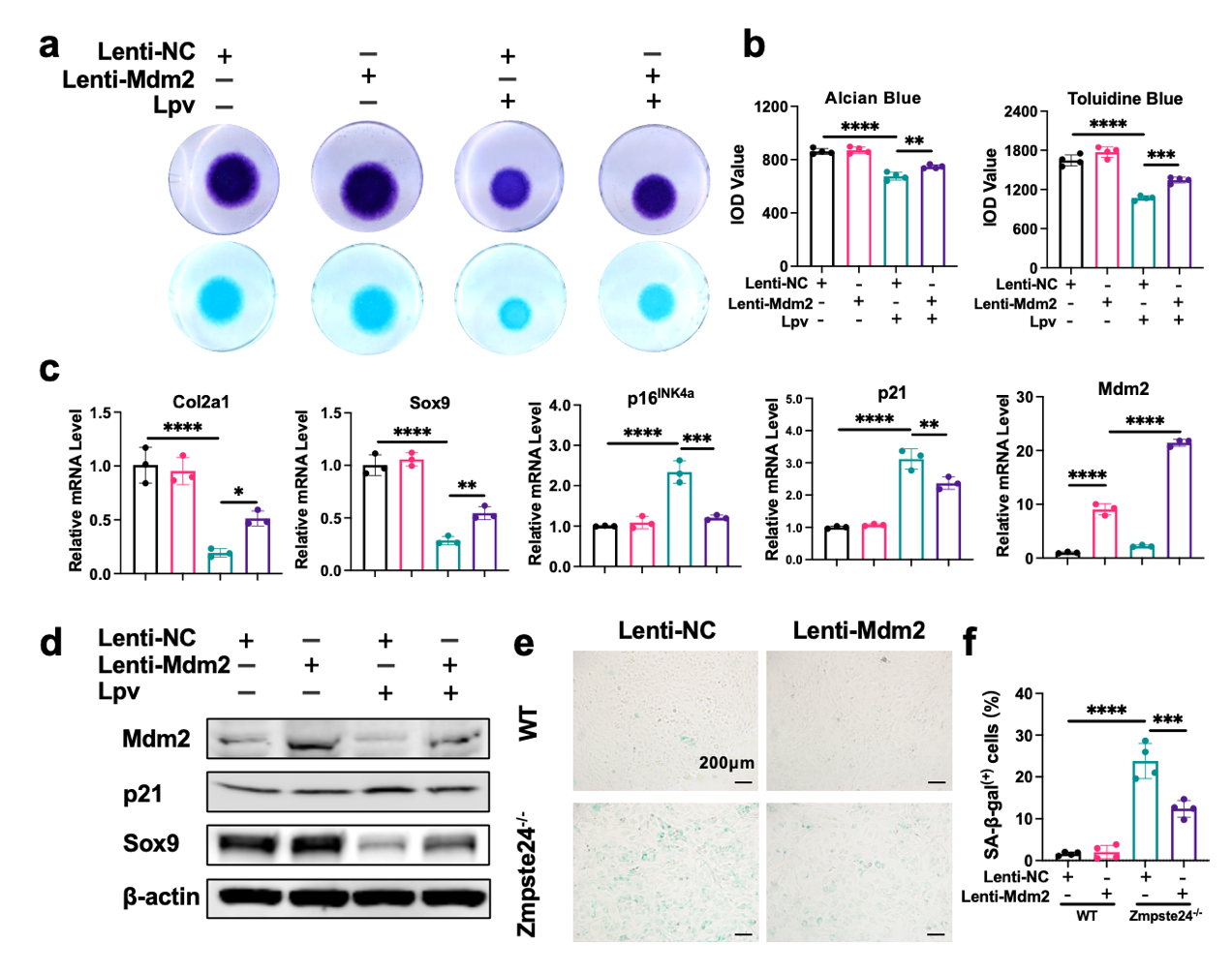
Supplementary Figure 14: Overexpression of Mdm2 can rescue Zmpste24 knockout-induced cartilage degeneration and senescence.**

(a, b) Alcian Blue and Toluidine Blue staining of micromass under Mdm2 overexpression and lopinavir treatment, with quantification. (c, d) mRNA and protein levels of Mdm2, Col2a1, Sox9, p16INK4a, p21, and p53 under Mdm2 overexpression and lopinavir treatment. (e, f) SA-β-gal staining and quantification under Mdm2 overexpression and lopinavir treatment. Scale bar: 200 μm. Data are shown as mean ± SD. *P<0.05, **P<0.01, ***P<0.001, and ****P<0.0001.
